# *Xenopus*: An *in vivo* model for imaging the inflammatory response following injury and bacterial infection

**DOI:** 10.1016/j.ydbio.2015.03.008

**Published:** 2015-12-15

**Authors:** Roberto Paredes, Shoko Ishibashi, Roisin Borrill, Jacques Robert, Enrique Amaya

**Affiliations:** a Faculty of Life Sciences, University of Manchester, Oxford Road, Manchester M13 9PT, United Kingdom; b The Healing Foundation Centre, University of Manchester, Oxford Road, Manchester M13 9PT, United Kingdom; c Department of Microbiology and Immunology, University of Rochester Medical Center, 601 Elmwood Ave, Rochester, NY 14642, USA

**Keywords:** Wound healing, Tissue regeneration, Inflammation, Neutrophils, macrophages, Live imaging, Leukocyte extravasation, Transgenic lines, *Mpeg1*, *Lurp1*

## Abstract

A major goal in regenerative medicine is to identify therapies to facilitate our body׳s innate abilities to repair and regenerate following injury, disease or aging. In the past decade it has become apparent that the innate immune system is able to affect the speed and quality of the regenerative response through mechanisms that are not entirely clear. For this reason there has been a resurgent interest in investigating the role of inflammation during tissue repair and regeneration. Remarkably, there have only been a handful of such studies using organisms with high regenerative capacity. Here we perform a study of the inflammatory response following injury in *Xenopus* larvae, which are able to achieve scarless wound healing and to regenerate appendages, as a preamble into understanding the role that inflammation plays during tissue repair and regeneration in this organism. We characterized the morphology and migratory behavior of granulocytes and macrophages following sterile and infected wounding regimes, using various transgenic lines that labeled different types of myeloid lineages, including granulocytes and macrophages. Using this approach we found that the inflammatory response following injury and infection in *Xenopus* larvae is very similar to that seen in humans, suggesting that this model provides an easily tractable and medically relevant system to investigate inflammation following injury and infection *in vivo*.

## Highlights

• *Xenopus* larvae is an ideal model to study injury-induced inflammation *in vivo.*
• *Xenopus* larvae provides an easily tractable model of human inflammation.
• *Xenopus* larvae provides a powerful model for investigating cell migration *in vivo.*

## Introduction

Multicellular organisms suffer a constant barrage of assaults in the form of injuries, which could lead to death from infection if not addressed quickly and efficiently. For this reason, selective pressures have led to the evolution of robust mechanisms aimed at healing wounds and fighting opportunistic infections. Given their ancient origins, it is not surprising that many of the mechanisms responsible for wound healing and fighting infections are highly conserved across the animal kingdom (Gurtner et al., 2008, Kimbrell and Beutler, 2001, Seifert and Maden, 2014, Sonnemann and Bement, 2011, Xu et al., 2012). However, despite their overall conservation it is nevertheless notable that some organisms are able to heal wounds better than others, and in addition, the capacity to heal wounds often change during the lifetime of the animal (Ferguson and O׳Kane, 2004, Seifert et al., 2012a, Seifert and Maden, 2014). For example, essentially all vertebrates, including humans, are capable of scarless wound healing as embryos, but this capacity is largely lost in adulthood (Ferguson and O׳Kane, 2004, Ud-Din et al., 2014). Intriguingly, some organisms, including some amphibians, are capable of regenerative scarless wound healing throughout their lifetimes (Godwin and Rosenthal, 2014, Seifert et al., 2012b). Thus, there is a significant amount of interest in elucidating the mechanisms that allow these organisms to achieve perfect regenerative skin repair, as a preamble to identifying novel therapies that might facilitate the same outcome in human patients (Godwin, 2014, Seifert and Maden, 2014).

Injury initiates a series of morphogenetic events enacted by the wound resident cells, culminating in wound closure (Gurtner et al., 2008, Sonnemann and Bement, 2011). An overlapping series of events following injury are performed by immune cells, comprising primarily neutrophils and macrophages (Eming et al., 2007). These cells mount an inflammatory response, which play several diverse roles during wound healing, including the removal of necrotic and apoptotic corpses as well as the elimination of invading bacteria, which could lead to potentially lethal infections. Although their role in fighting infection has been appreciated for a long time, only in the last two decades has it become apparent that inflammatory cells have additional diverse and important roles during tissue repair and regeneration (Martin and Leibovich, 2005). For example, both neutrophils and macrophages have been implicated in modulating the speed and/or quality of wound healing (Dovi et al., 2003, Dovi et al., 2004, Martin et al., 2003). They have also been shown to provide essential roles during the regeneration of various tissues, organs and appendages (Chazaud, 2014, Godwin et al., 2013, Li et al., 2012, Petrie et al., 2014, Tidball and Wehling-Henricks, 2007). Inflammatory cells are now recognized to consist of a diverse population of cells, some of which provide beneficial roles and others detrimental roles during wound healing and regeneration. For this reason there has been a resurgent interest in understanding the various roles that particular myeloid lineages may have during wound healing and indeed, whether manipulating the type of inflammatory response may facilitate wound healing and regeneration in human patients (Wicks et al., 2014). Intriguingly, while amphibians are capable of achieving scarless wound healing, very little is known about the nature of the inflammatory response following injury in amphibians or which types of inflammatory cells are recruited to wounds during healing in these organisms. *Xenopus* embryos and larvae provide useful models for investigating the fate and behavior of myeloid cells *in vivo* after injury using time-lapse video microscopy (Chen et al., 2009, Costa et al., 2008).

In this study we used *Xenopus laevis* transgenic lines, which label different subsets of myeloid lineages in larvae with different colored fluorescent proteins, to investigate the inflammatory response following injury using *in vivo* time-lapse imaging. In this way, we were able to analyze the distribution, motility and migratory behavior of macrophages and granulocytes toward wounds and/or localized bacterial infections.

## Materials and methods

### Transgenic animals

*Xenopus laevis* transgenic lines were generated using the Restriction Enzyme Mediated Integration (REMI) technique (Amaya and Kroll, 2010, Kroll and Amaya, 1996). Zebrafish *mpeg1* gene promoter driving GFP or mCherry were kindly donated by Graham Lieschke (Ellett et al., 2011) and the *lurp1*:GFP transgenic line was kindly donated by Stuart Smith and Timothy Mohun (Smith et al., 2002). Tadpoles at developmental stage 46–50 were used (3 weeks of age). All animal experiments were covered by a UK Home Office Project License.

### Microscopy and cell shape analysis

Imaging acquisitions were made in a FluoView1000 confocal microscope system (Olympus). We processed the fluorescent channel acquisitions as z-stack projections and the transmitted light channel acquisition as the best-*in focus* plane. The normalized lamellipodia area (NLA) was calculated as the ratio of the whole cell area (WCA) over the cell body cell area, using a modification of a previously described method (Evans et al., 2013). To determine the whole body area and the cell body area, different fluorescence thresholds using the color threshold plug-in in ImageJ were created, as the lamellopodia exhibit lower fluorescence intensity than the cell body (Abramoff et al., 2004)].

### Wounding assays and *in vivo* cell tracking

Stage 46 or older transgenic *X. laevis* larvae with fluorescently labeled myeloid cells were anesthetized with a 0.01% tricaine (MS-222, Sigma) in 0.1× MMR solution and then mounted in a viscous solution [0.05% tricaine, 0.1× MMR, 2% methylcellulose (Sigma)] over a glass-bottomed Petri dish (MatTek). After mounting, the middle region of ventral fin was wounded using either a Dumont #55 Forceps (Fine Scientific Tool) or a 0.3 mm diameter surgical biopsy punch (INEX, 60540 Puiseaux-le-Hauberger, France). Alternatively, the distal third of the tail was amputated with a surgical blade (scleral knife, FEATHER Safety Razor Co., Ltd., Japan). To produce infected wounds a biopsy puncher was scraped over several colonies of *E. coli* on an agar-plate prior to wounding. All the procedures were carried out under a Leica S8APO dissecting microscope. In the case of single cell ablations, a melanophore was irradiating with a continuous laser pulse for 1 min [10 mw multi-Argon Laser, FlouView1000 confocal microscope system (Olympus)]. 4D-time lapse images were acquired in a FlouView1000 confocal microscope system (Olympus) as 10 confocal planes (covering between 120 and 240 μm depth) each 2 min (blocks of 2 h, up 10 h of recording). Each time point consisting of 10 confocal planes were flattened down to create a continuous video projection (ImageJ software). The resultant time lapse movies were analyzed with the Mtrack2 plug-in of the ImageJ software to compute the *x*,*y* Cartesian coordinates of each individual migrating cell path. Polar coordinates, vectorial information and the circular statistics analysis of the migrating cell vectors were calculated with CircStat Toolbox of MatLab 2009a software (The MathWorks, Inc.) (Berens, 2009). In some cases various anatomical features were highlighted, such blood vessels, capillary networks, muscle fibers or intersomitic spaces. To achieve this, false-colored masks of the relevant anatomical feature were superimposed over the best in focus image taken as a reference in the bright field channel (Opacity tool, Adobe Illustrator CS4).

## Results

### Assessment of the migratory behavior of myeloid cell populations in unwounded *Xenopus* larvae

**Fig. 1:**
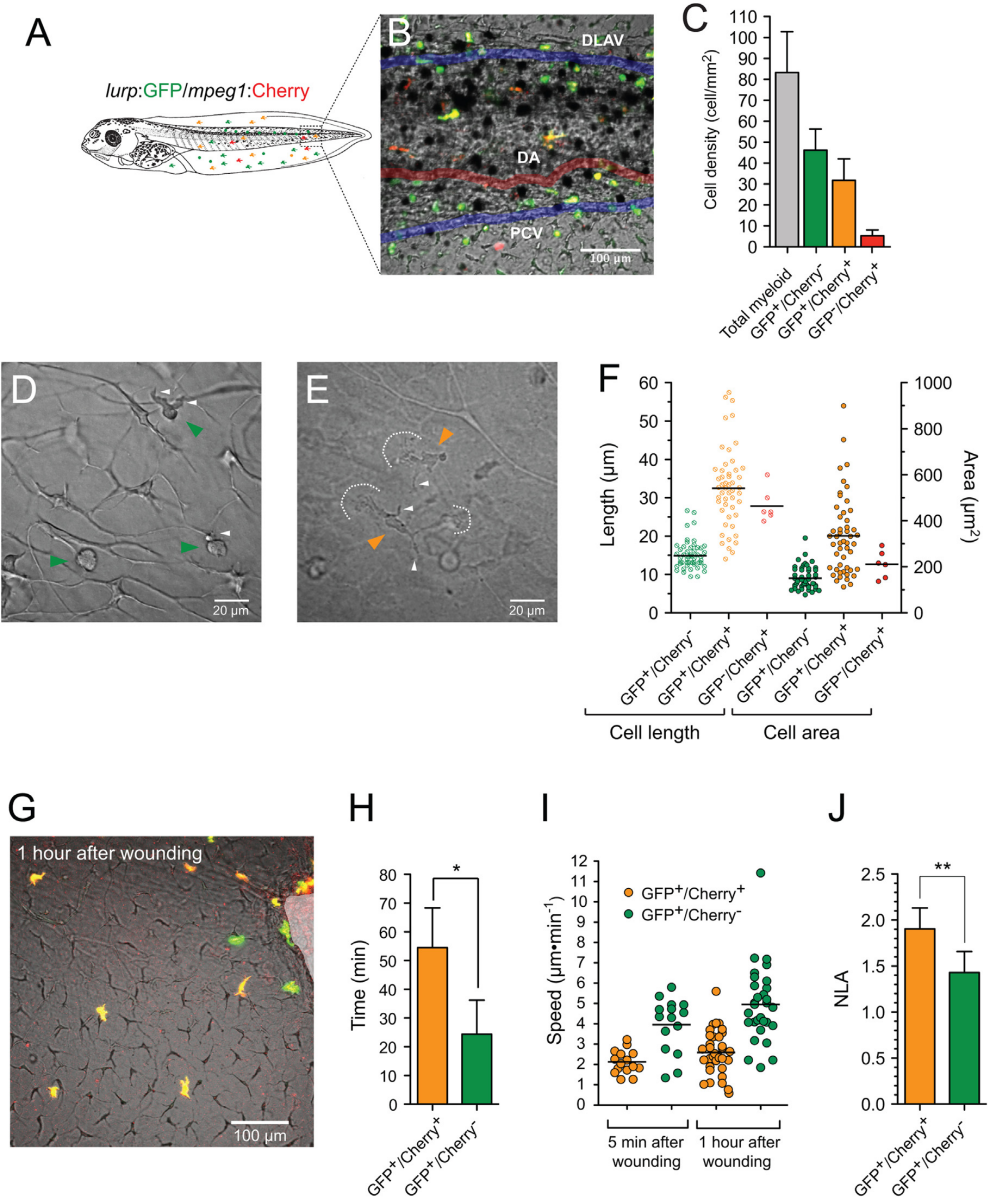
Heterogeneity of myeloid cell populations in *X. laevis* larvae. (A) Schematic representation of a double transgenic *Xenopus* larva expressing Green Fluorescent Protein (GFP) under the control of the *Xenopus lurp1* gene promoter and the mCherry Fluorescent Protein (mCherry) under the control of zebrafish *mpeg1* gene promoter. Myeloid cells are differentially labeled according to GFP or mCherry expression. (B) Dual channel confocal projections merged with the best in focus transmitted light image. Granulocyte-like cells, labeled in green are located all along the larval body. Macrophage-like cells appeared labeled in yellow or red due the mCherry expression driven from the *mpeg1* promoter. The labeled myeloid cells are located in scattered positions along the tail and in close proximity to the major blood vessels, such as the dorsal aorta (DA, colored red mask), the posterior cardinal vein (PCV, colored blue mask) and the dorsal longitudinal anatomizing vessel (DLAV, colored blue mask). (C) Myeloid cell subpopulations density was calculated by counting each cell labeled group by mm^2^ in different larvae (*n*=5). (D) and (E) Transmitted light images of the ventral fin of *Xenopus* larvae. *lurp*+/*mpeg*− (Granulocyte-like) and *lurp*+/*mpeg*+ macrophage-like cells are denoted by green and orange arrowheads, respectively. Cellular projections are denoted with arrowheads and dashed lines. See Video 3, Video 4, Video 5, associated with panels D and E. (F) Cell length was calculated between the most separated end points of each cell and cell area was calculated by creating masks of the fluorescent expression of each cell (ImageJ software). (G) Dual channel confocal projection merged with the best in focus transmitted light image. A small wound was inflicted in the ventral fin to activate myeloid cell migration (white overshadow at the right hand site). See supplementary video 6, associated with this panel. (H) Time of activation was calculated from the moment the wound was made and when the cells started to migrate (continuous changes in position of more than one cell body size) for the given subpopulation in 5 different larvae. *lurp*+/*mpeg*+ cells (macrophage-like) are denoted in orange and *lurp*+/*mpeg*− cells (granulocyte-like) are denoted in green. Mann–Whitney *U*-test, * *p*<0.05. (I) Speed of migration was calculated by tracking individual cells during a 1-h time block as total path length by time. (J) Normalized Lamellipodia Area (NLA) was calculated as the ratio between the whole cell area and the cell body area (area mask generated in ImageJ software). Mann–Whitney *U*-test, *n*=8, **p<0.01.

**Fig. 2:**
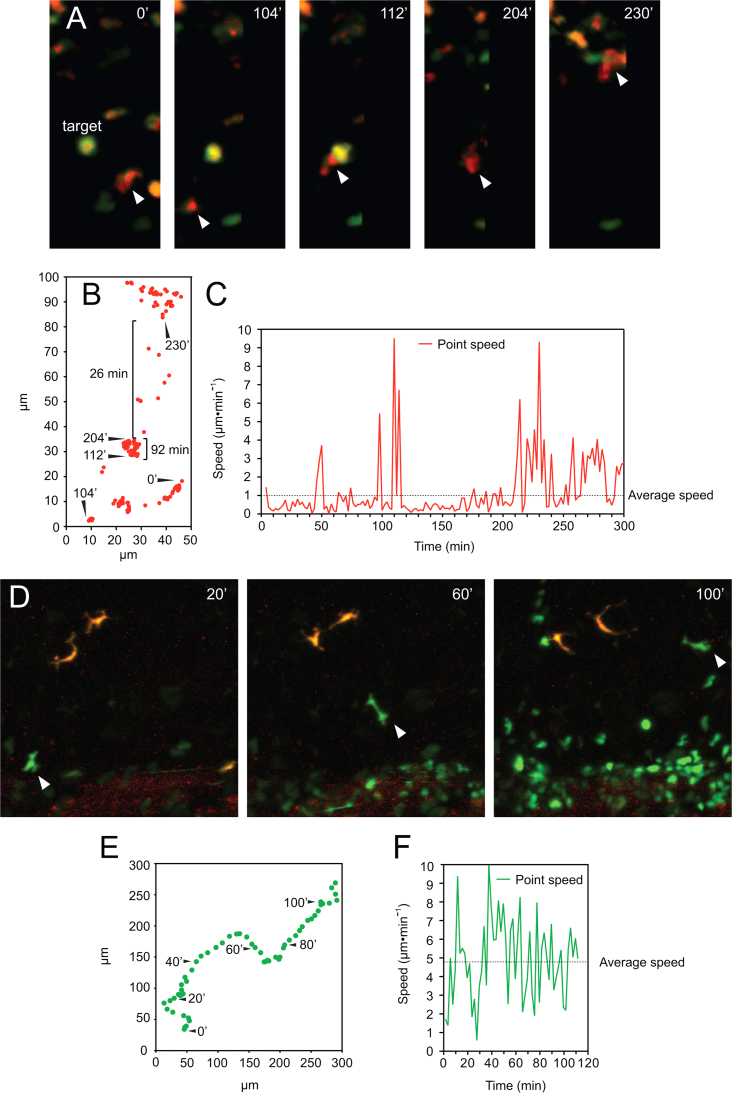
Tracing myeloid cell migration. (A) Representative still images of dual channel confocal projection time-lapse microscopy, following one *mpeg*+ macrophage-like cell (white arrowhead). A target cell (a necrotic/apoptotic GFP+ granulocyte), which is engulfed by a macrophage, is denoted and the time of the sequence frames is labeled at the top right corner. See supplementary video 7, associated with this panel. (B) Single cell tracking of the *mpeg*+ cell from panel A was made with the Mtrack2 plug-in for ImageJ. Time points were acquired each 2 min and the Cartesian coordinates of the track were plotted as red dots. Time point counterparts with the panel A are indicated. (C) Point speed of migration (red line) was calculated for each time point and the average speed through the time lapse is denoted as a dashed line. (D) Representative still images of dual channel confocal projection time lapse pointing at a *lurp*+ granulocyte-like cell (white arrowhead). An excisional wound was made at the top right corner and the time labels denote minutes after wounding. See supplementary video 8, associated with this panel. (E) Single cell track data was extracted from the time lapse showed in panel D and time point labels are added as guidance. (F) Point speed of migration (red line) was calculated for each time point and the average speed through the time lapse is denoted as a dashed line.

As in other vertebrate models (Hartenstein, 2006), myeloid cells with morphology typical of granulocytes/neutrophils and monocyte/macrophages have been described in *Xenopus* adults and larvae (Chen et al., 2009, Claver and Quaglia, 2009, Hadji-Azimi et al., 1987, Lehman, 1953, Morales et al., 2010). To further investigate whether this heterogeneity of myeloid cell types found in *Xenopus* tadpoles correspond to heterogeneity in motility and behavior we first conducted a series of time-lapse microscopic observations on intact transgenic animals, expressing fluorescent proteins within their myeloid lineages. For this purpose we used the *lurp1*:GFP transgenic line that expresses GFP in most myeloid cells (Smith et al., 2002). In addition, we generated *Xenopus* transgenic larvae expressing either GFP (green) or mCherry (red) under the control of the zebrafish *mpeg1* promoter, which is predominantly expressed in macrophage lineages (Ellett et al., 2011). We also generated double *mpeg1*:mCherry and *lurp1*:GFP trangenic embryos expressing both markers (Fig. 1). As expected, *lurp1*:GFP/m*peg1*:mCherry double transgenic *Xenopus* tadpoles contained a subset of myeloid cells expressing GFP only, which corresponded to neutrophils (Fig. 1A and B). In addition, we identified another subset of myeloid cells that expressed both GFP and mCherry simultaneously, and thus appeared yellow (Fig. 1A and B). The latter cells displayed the morphology of macrophages. However, we were surprised to see a third subset of cells with monocytic morphology expressing mCherry only (Fig. 1A and B). This mCherry-only subset of cells accounted for 5% of the fluorescent myeloid cells within the tail tissue (Figs. 1C and 2A). The tissue resident lurp+ fluorescent myeloid cells were generally present in close proximity to the major blood vessels in the trunk of the tail (dorsal aorta, posterior cardinal vein and the posterior longitudinal anastomosing vessel) and also they were found in scattered positions along the dorsal and ventral fins (Fig. 1B). Time-lapse observations of the *lurp1*:GFP transgenic larvae along the ventral fin revealed a subset of myeloid cells which were relatively quiescent, and another subset of myeloid cells with more pronounced motility. Many of these cells were localized inside blood vessels, rolling along the endothelium layer (Supplementary Video 1, Video 2).

By compiling traces of hundreds of cells from the 3 different transgenic lines (single and double transgenic ones), we could distinguish two main types of motility behaviors related to distinct cell morphologies: (1) neutrophil-like small round compact cells (15 μm average length, 150 μm^2^ average cell area) that projected single or fewer than 3 lamellipodia projections in the direction of movement (Fig. 1D and F; and Supplementary video 3); and (2) macrophage-like larger and flatter cells (32 μm average length and 330 μm^2^ average cell area) that projected multiple lamellipodia (more than 3) in several directions (Fig. 1E and F; and Supplementary video 4 and video 5).

### Motility behaviors of labeled myeloid cell populations following injury in *Xenopus* larvae

We next analyzed the migratory response of the different populations of myeloid cells following wounding in the ventral fin of *lurp1*:GFP/*mpeg1*:mCherry transgenic *X. laevis* larvae (Fig. 1G and supplementary video 6). Neutrophil-like (lurp+/mpeg−) cells within a radius of 100 μm from the site of injury responded by moving toward the wound within the first 20 min following injury, whereas the macrophage-like (lurp+/mpeg+) cells moved toward the wound up to an hour later (Fig. 1H). Furthermore, cell tracking analyses revealed that the monocyte/macrophage population migrated with a slower average speed compared to cells with neutrophil morphology, expressing GFP only (Fig. 1I). In addition to their faster motility, lurp+/mpeg− neutrophil-like cells adopted a shape distinct from the macrophage-like population while migrating. To quantify these observations we calculated the normalized lamellipodia area (NLA) as the ratio of the whole cell area over the cell body cell area. We found that the lurp+/mpeg+ monocytic/macrophage like cells projected larger lamellipodia area than lurp+/mpeg− granulocytic like cells (Fig. 1J).

To substantiate the distinctive behavior of the putative macrophages and granulocytes we examined single-cell tracks by 4-D fluorescence video microscopy in *lurp*:GFP/*mpeg1*:Cherry transgenic larvae (Fig. 2A; and Supplementary video 7). Time-lapse images were acquired every 2 min and we used the algorithm MTrack2 in ImageJ (NHI, Bethesda, MD) over threshold binary images to obtain the path of migration of selected cells through time. As the cell size ranged from 10 to 60 μm (Fig. 1F) we set minimum and maximum cut offs to discriminate individual cells from any debris or cell aggregates. The software tracked moving objects that fulfilled the following criteria: appropriate size range; continuous presence throughout the selected time frames; and no jump more than 2 body sizes between one time point and the next one. The tracking output was a collection of (*x*,*y*) positional coordinates (Cartesian coordinates) at all the times points for each cell tracked (Fig. 2B and E). The length of the path and the net displacement were also given. We calculated the instantaneous speed and the average speed for individual cells (Fig. 2C and F, solid lines and dashed lines respectively). With this analysis, we found that the lurp−/mpeg+ macrophages are phagocytic (Fig. 2A, white arrowheads, and supplementary video 7). Furthermore, lurp−/mpeg+ macrophages had slow average speed of migration with high peak of instantaneous speed, associated with “target chasing”, for example, a necrotic/apoptotic lurp+ neutrophil/granulocyte near the wound, 24 h after injury. (Fig. 2C). In contrast, lurp+/mpeg− neutrophils/granulocytes (Fig. 2D, white arrowheads; Supplementary video 8) showed significantly higher spatial displacement (Fig. 2E) and higher average speed than macrophages (Fig. 2F).

**Fig. 3:**
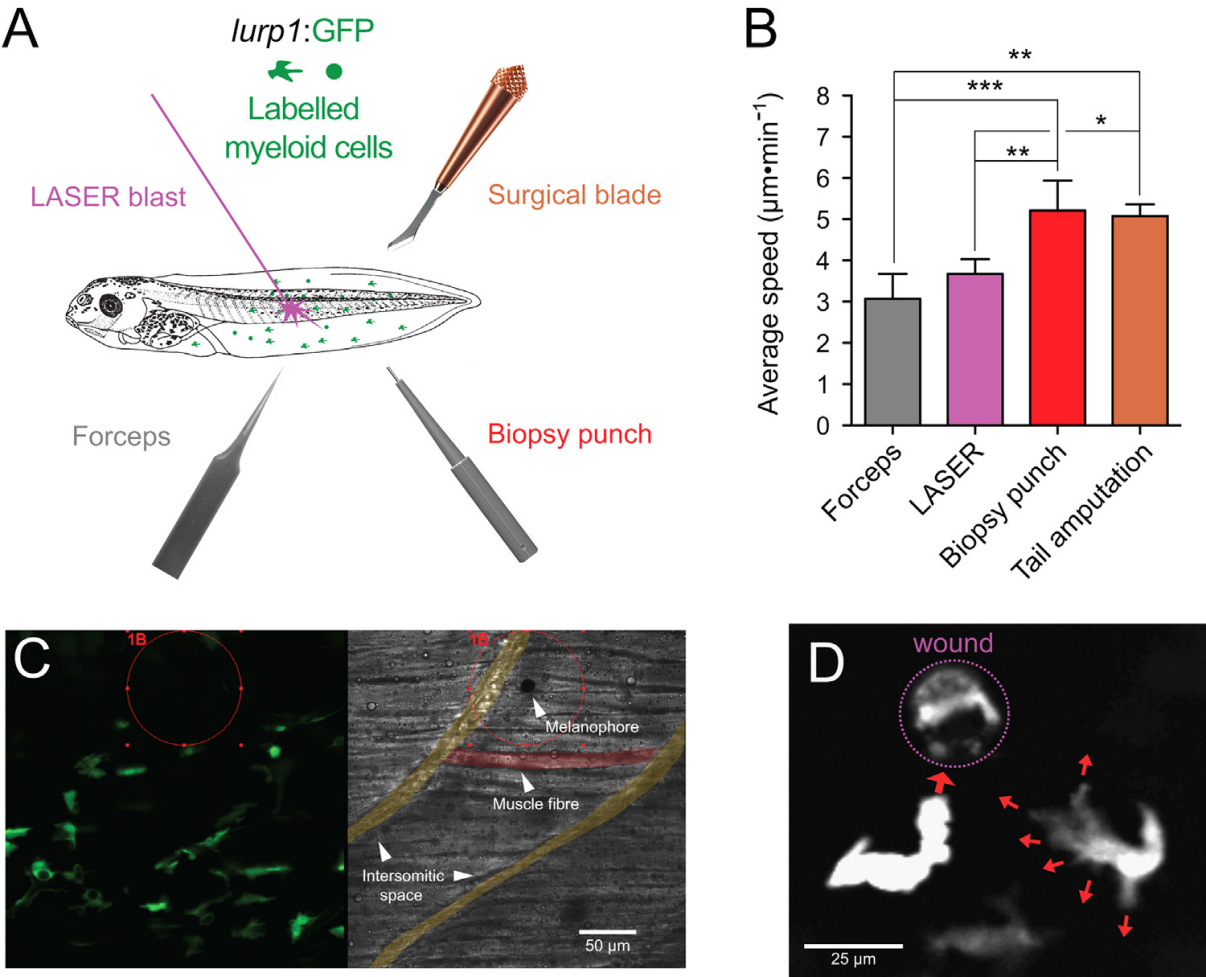
Real-time responses of myeloid cells to mechanical injury. (A) Schematic representation of the different wounding methods used on transgenic *X. laevis* larvae. (B) Average speed was calculated from the *lurp*+ cell population in at least 3 different experiments for each condition 1 h after wounding. One-way analysis of the variance (ANOVA) and Tukey׳s Multiple Comparison Test were used for statistical analysis. *p<0.05; **p<0.01; ***p<0.001. (C) Single channel confocal projection and best in focus transmitted light images after melanophore ablation injury. The LASER pulse was directed at the area denoted 1B (red circle). The melanophore (red circle), the intersomitic space (colored yellow mask) and some muscle fibers (colored red mask) are highlighted for reference. See supplementary video 9, associated with this panel. (D) Single channel confocal projection (gray scale) of adjacent myeloid cells migrating towards the wound stimulus, projecting lamellipodia denoted by red arrows.

To obtain additional evidence of the distinctive behavior of granulocytes and monocytes upon injury, we tested four independent types of localized sterile lesions in the fins of *lurp1*:GFP transgenic larvae: (1) 0.05 mm×0.02 mm tip-forceps pin prick; (2) 0.02 mm in diameter melanophore LASER ablation; (3) 0.3 mm biopsy punch excisional wound; and (4) surgical blade tail amputation (Fig. 3A). All wounding assays tested produced a rapid response in the adjacent myeloid cells. For instance, LASER damage of a melanophore (Fig. 3C, red circle; Supplementary video 9) was sufficient to activate the surrounding myeloid cells within 1 h at 20 °C. Nevertheless, cells activated by small wounds, such as piercing with forceps or LASER ablation, exhibited lower average speed of migration toward the wound, compared to the speed of migration induced by larger wounds, such as biopsy punch excisional wounds or tail amputation (Fig. 3B). Although, GFP was expressed by most myeloid cells, we could distinguish granulocyte-like cells that displayed a fewer number of lamellipodial projections in the direction of movement (Fig. 3D, left cell), from macrophage-like cells presenting multiple lamellipodia projections (Fig. 3D, right cell). This finding confirmed that the faster moving neutrophil-like cells and the slower moving monocytic-like cells shown in Fig. 1J were related to the morphology of their cellular projections.

**Fig. 4:**
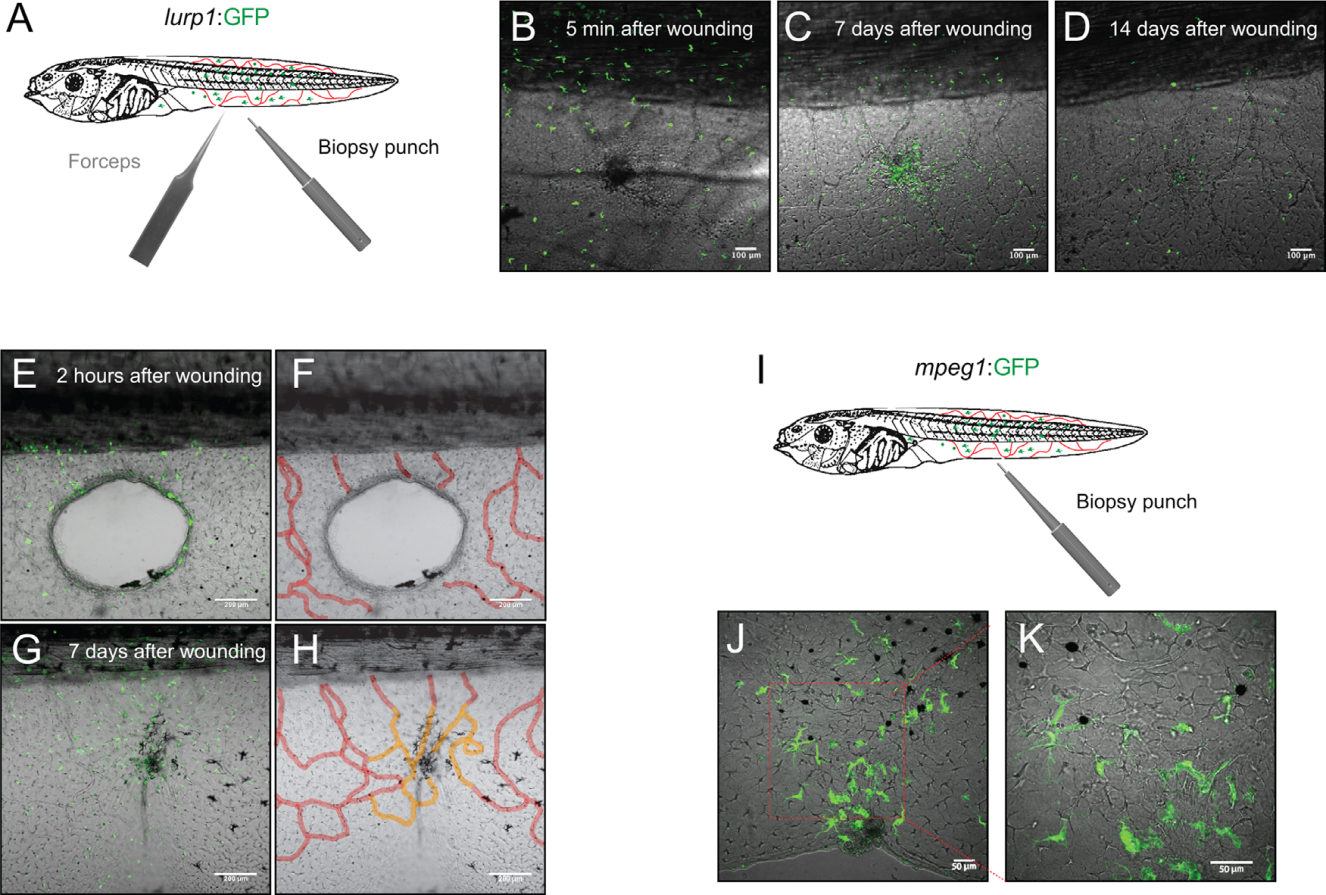
Wound healing after injury in *Xenopus* larvae. (A) Schematic representation of the forceps piercing and biopsy punch wound assays used in transgenic *lurp1*:GFP *X. laevis* larvae. Myeloid cells are labeled in green and fin capillary network is labeled in red. (B)–(D) Single channel confocal projection and best in focus transmitted light image merge of a forceps pierced wound (dark area at the center) at 5 min, 7 days and 14 days after wounding, respectively. (E) and (F) Single channel confocal projection and best in focus transmitted light image merge of a biopsy punch wound (excisional wound at the center) at 2 h after wounding. GFP+ cells are labeled myeloid cells (E) and the capillary network is highlighted with a red mask. (G) and (H) Single channel confocal projection and best in focus transmitted light image merge of the healed wound 7 days after injury. GFP+ cells are labeled myeloid cells (G). The capillary network is highlighted with a red mask (old blood vessels) and an orange mask (new blood vessels). (I) Schematic representation of a biopsy punch wounding assays in transgenic m*peg1*:GFP *X. laevis* larvae. (J) and (K) Single channel confocal projection and best in focus transmitted light image merge of a healed wound 8 days after injury. GFP+ cells are labeled macrophage-like cells. The excisional wound was originally at the center of the field.

*Xenopus* larvae are able to undergo scarless wound healing and regenerate their tails within a week after injury (Love et al., 2013). We tested the ability of tadpoles to heal puncture or large excisional wounds, and we found that they are able to fully heal these wounds within 7 days of injury (Fig. 4). As noted before, both of these types of injury resulted in recruitment of myeloid cells to the site of injury, but these eventually left the healed area by about 2 weeks (Fig. 4). In excisional wounds that cut through blood vessels (Fig. 4F, red mask), the healing process resulted in a full restoration of the capillary network within a week following injury (Fig. 4H, orange mask). Macrophage-like (mpeg+) cells persisted at the wound site for at least a week after injury (Fig. 4J and K). We also observed the restoration of melanophores at the wound site following wound healing (Fig. 4D, G and J).

**Fig. 5:**
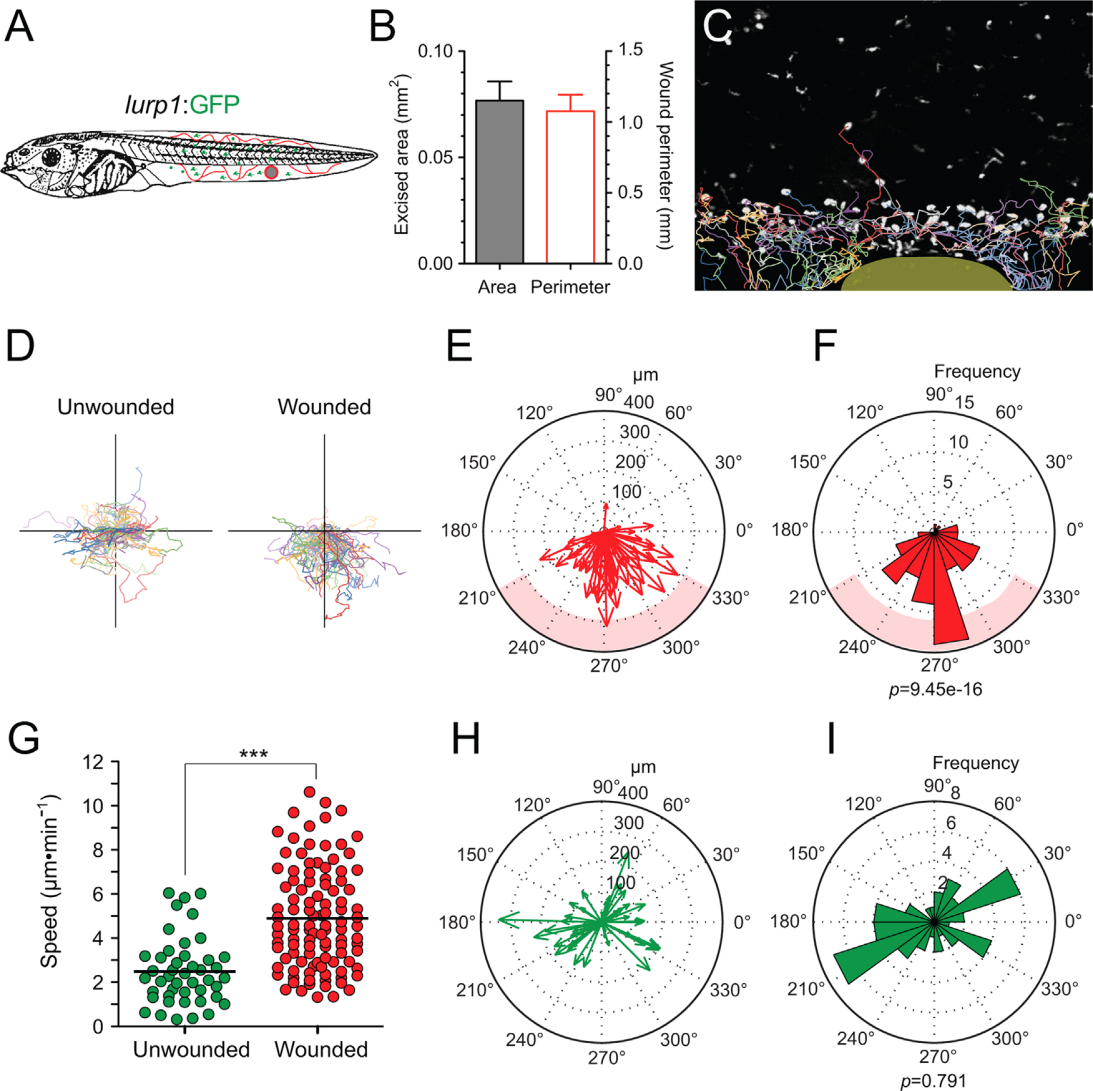
Myeloid cells migrate preferentially towards the wound site after injury. (A) Schematic representation of a biopsy punch wound assay in *lurp1*:GFP transgenic *Xenopus* larva *in vivo*. The perimeter (red circle) and the area (gray circle) of the excisional wound are indicated. (B) Averages of the area (gray bar) and the perimeter (red line bar) of the wounds measured from 3 independent experiments. (C) One channel confocal projection of the starting point of migration of the myeloid GFP+ cells (gray scale) merged with the cell tracks obtained from the time-lapse analysis. The wound site is highlighted in yellow. See supplementary video 10, associated with this panel. (D) The cell tracks were plotted forcing a common origin in a quadrant mesh. (E) and (F) The magnitude and the angles of the displacement vectors obtained from the GFP+ cell tracks after biopsy punch wounding assay were plotted in rose charts (MatLab). The location of the wound site is denoted by a pink overshadow. Rayleigh test (CircStat for MatLab, The MatWorks) *p*-value is also indicated. (G) The speed of migration was calculated for each GFP+ cell following light stimulation (unwounded, green dots) or after a biopsy punch-wounding assay (wounded, red dots). The mean speed is indicated with a transversal black line. Mann–Whitney *U*-test, ***p<0.001. (H) and (I) The magnitude and the angles of the displacement vectors obtained from the GFP+ cell tracks after light stimulation were plotted in rose charts (MatLab). Rayleigh test (CircStat for MatLab, The MatWorks) *p*-value is also indicated.

**Fig. 6:**
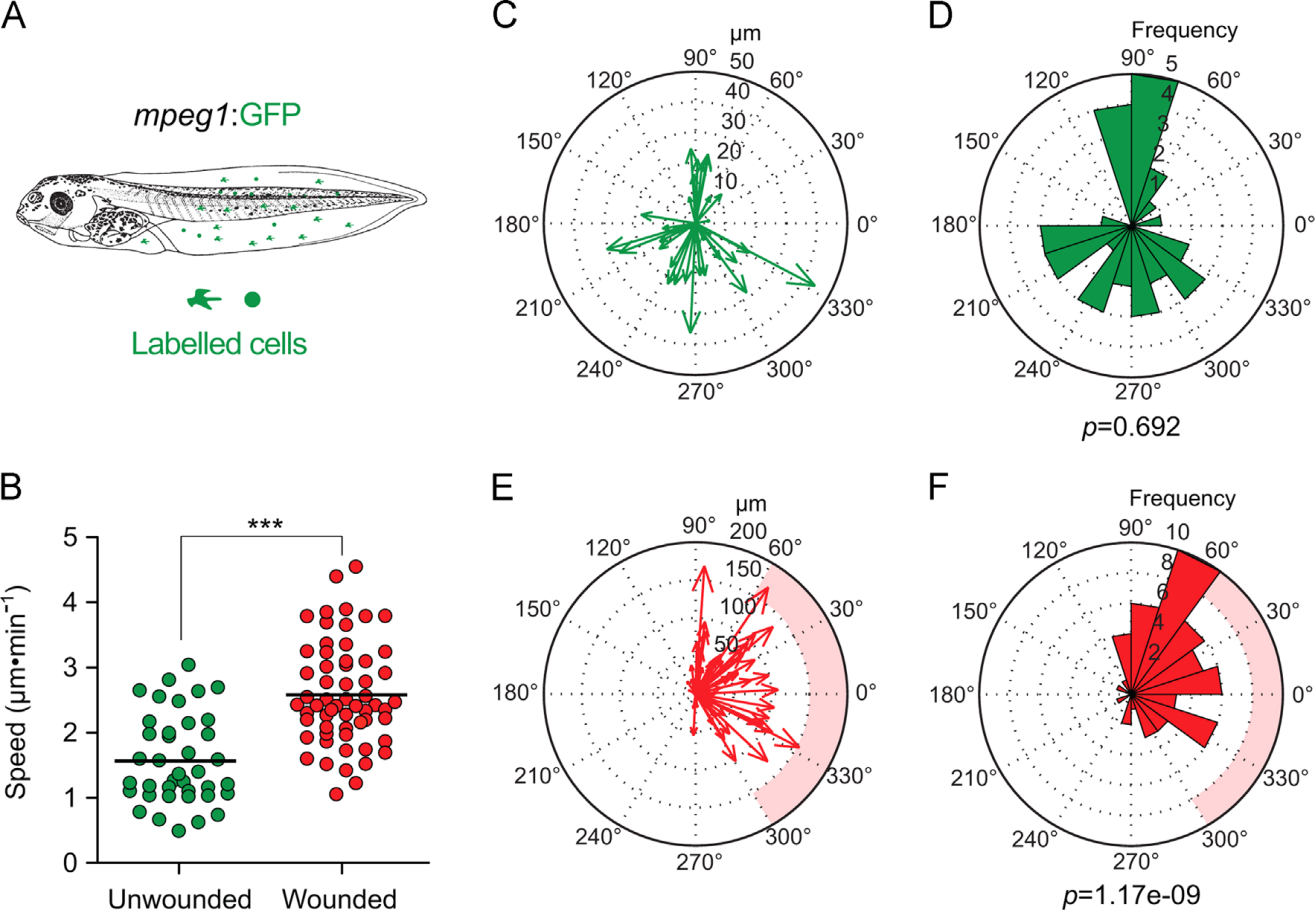
Macrophage-like cells migrate preferentially towards the wound site after injury. (A) Schematic representation of a *mpeg1*:GFP transgenic *Xenopus* larva. (B) The speed of migration was calculated for each GFP+ cell following light stimulation (unwounded, green dots) or after biopsy punch wounding (wounded, red dots). The mean speed is indicated with a transversal black line. Mann–Whitney *U*-test, ***p<0.001. (C) and (D) The magnitude and the angles of the displacement vectors obtained from the GFP+ cell tracks after light stimulation were plotted in rose charts (MatLab). (E) and (F) The magnitude and the angles of the displacement vectors obtained from the GFP+ cell tracks after biopsy punch wounding were plotted in rose charts (MatLab). The location of the wound site is denoted by a pink overshadow. Rayleigh test (CircStat for MatLab, The MatWorks) *p*-values are also indicated.

To determine whether injury also alters the direction of myeloid cell migration *in vivo*, we used the reliable biopsy-punch wounding assay on *lurp1*:GFP transgenic *Xenopus* larvae (Fig. 5A). A round excisional wound was inflicted in the middle section of the ventral fin. We tested wound consistency by measuring the area of the excised tissue and the perimeter of the wound site (Fig. 5B). The GFP+ myeloid subpopulations were traced in real time by generating paths of migration (Fig. 5C; Supplementary video 10) as described previously. To address if there was a difference in the direction of migration, we compared the mechanical injury (biopsy punch) to a uniform light stimulation (repetitive 5 s pulses of 488 nm LASER each two minutes for 2 h). We found that mechanical injury resulted in a massive recruitment of myeloid cells towards the wound site within an hour after injury (Fig. 5D, right). In contrast, light stimulation activated cell migration only after 2 h (data not shown) and cells migrated without directional preference (Fig. 5D, left). We calculated the vectorial displacement by transforming the Cartesian coordinates into polar coordinates and the direction of movement was plotted in a compass or rose plots allocated within a 360° circular scale (MatLab 2009a, The MathWorks, Inc). For this particular example, the wound site is located at 270° and is labeled as pink shadows at the compass and rose plots. We also calculate the distribution of the angles of displacement (Fig. 5E and F). The Rayleigh test (CircStat for MatLab, The MatWorks) was applied to ask whether the angles distribution follows uniform distribution. A *p*-value less than 0.05 (*p*<0.05) indicates a significant departure from uniform distribution. Following light stimulation, we found a *p*-value of 0.79 indicating a uniform distribution of the angles of the displacement vectors, in other words, cells moved without a preferred direction (Fig. 5H and I). However, in the instance following a biopsy punch wound, a *p*-value of 9.45e−16 indicates a high confidence in the departure from uniformity (rejection of the null hypothesis), which in this particular case was in the direction of the wound located at 270° (Fig. 5E and F). We confirmed that the average speed of migration was significantly higher after wounding (Fig. 5G). The preferred direction of migration after wounding was observed for the two main myeloid populations, neutrophils and macrophages, as evidenced by similar experiments performed with the *mpeg1*:GFP transgenic line (Fig. 6), More specifically, *mpeg1*;GFP+ macrophage-like cells migrated towards the wound site (Fig. 6E and F) compared to the light stimulation alone controls (Fig. 6C and D). Their migration was also faster after wounding, relative to the unwounded scenario (Fig. 6B).

**Fig. 7:**
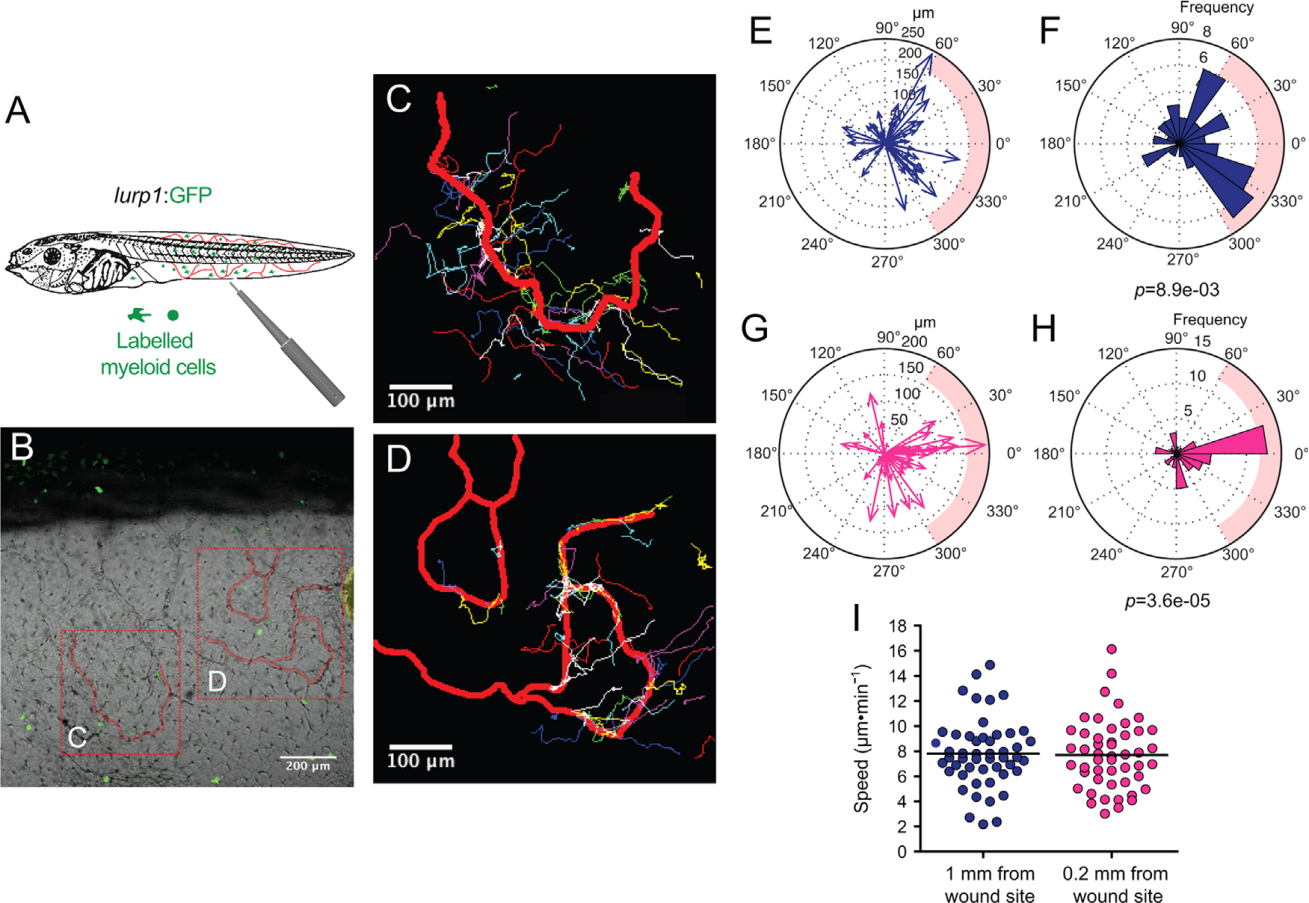
Leukocyte extravasation of myeloid cells in response to mechanical wounds. (A) Schematic representation of a biopsy punch wound assay and the capillary network (in red) at the ventral fin of a *lurp1*:GFP transgenic *Xenopus* larva. (B) One channel confocal projection merged with a transmitted light image of the ventral fin of a *lurp1*:GFP transgenic *X. laevis* larva. GFP+ myeloid cells appear in green. The wound edge at the right hand site is highlighted in yellow. The regions analyzed are denoted by red dashed squares. The capillaries are highlighted in red. See Video 11, Video 12, associated with this panel. (C) and (D) Path length reconstitution from the GFP+ cell tracking migrating from capillaries after wounding. A red mask indicating the capillary network is show as reference. (E) and (F) The magnitude and the angles of the displacement vectors obtained from the GFP+ cell tracked from the farthest imaged capillary from the edge of the wound were plotted in rose charts (MatLab). The location of the wound site is denoted by a pink overshadow. Rayleigh test (CircStat for MatLab, The MatWorks) *p*-value is also indicated. (G) and (H) The magnitude and the angles of the displacement vectors obtained from the GFP+ cell tracked from the closest imaged capillary from the wound edge were plotted in rose charts (MatLab). The location of the wound site is denoted by a pink overshadow. Rayleigh test (CircStat for MatLab, The MatWorks) *p*-value is also indicated. (I) The speed of migration was calculated for each GFP+ cell that extravasated from the farthest (blue dots) and closest (pink dots) of the edge of the wound. The mean speed is indicated with a transversal black line.

As described previously (*i.e.* Supplementary Video 1, Video 2), many *lurp1*:GFP+ myeloid cells are located within the lumen of blood vessels and these cells can extravasate into interstitial tissues. To determine whether the circulating GFP+ labeled myeloid cells could undergo diapedesis and be recruited to the injury tissue site, we performed biopsy punch wounds on *lurp1*:GFP transgenic larvae (st 50), where the fin vasculature was well developed (Fig. 7A–D). Extravasation of GFP+ cells occurred within 2 h after wounding from capillaries present in the vicinity of the wound site (within 1 mm) (highlighted in yellow toward the right hand side in Fig. 7B). GFP+ myeloid cells left the blood vessels in several directions, especially in the capillary located farther from the wound site (Fig. 7C; Supplementary video 11), Nevertheless, these cells rapidly gained directionality towards the injury stimuli (Fig. 7E and F). Myeloid cells coming from capillaries closer to the wound site (Fig. 7D; Supplementary video 12) migrated towards the wound site (Fig. 7G and H). We found no difference in the average speed between the labeled cells recruited from capillaries in the proximity of the wound compared to cells migrating from more distant capillaries (Fig. 7I).

### Response of larval mpeg+ monocytic cells to infected wounds

**Fig. 8:**
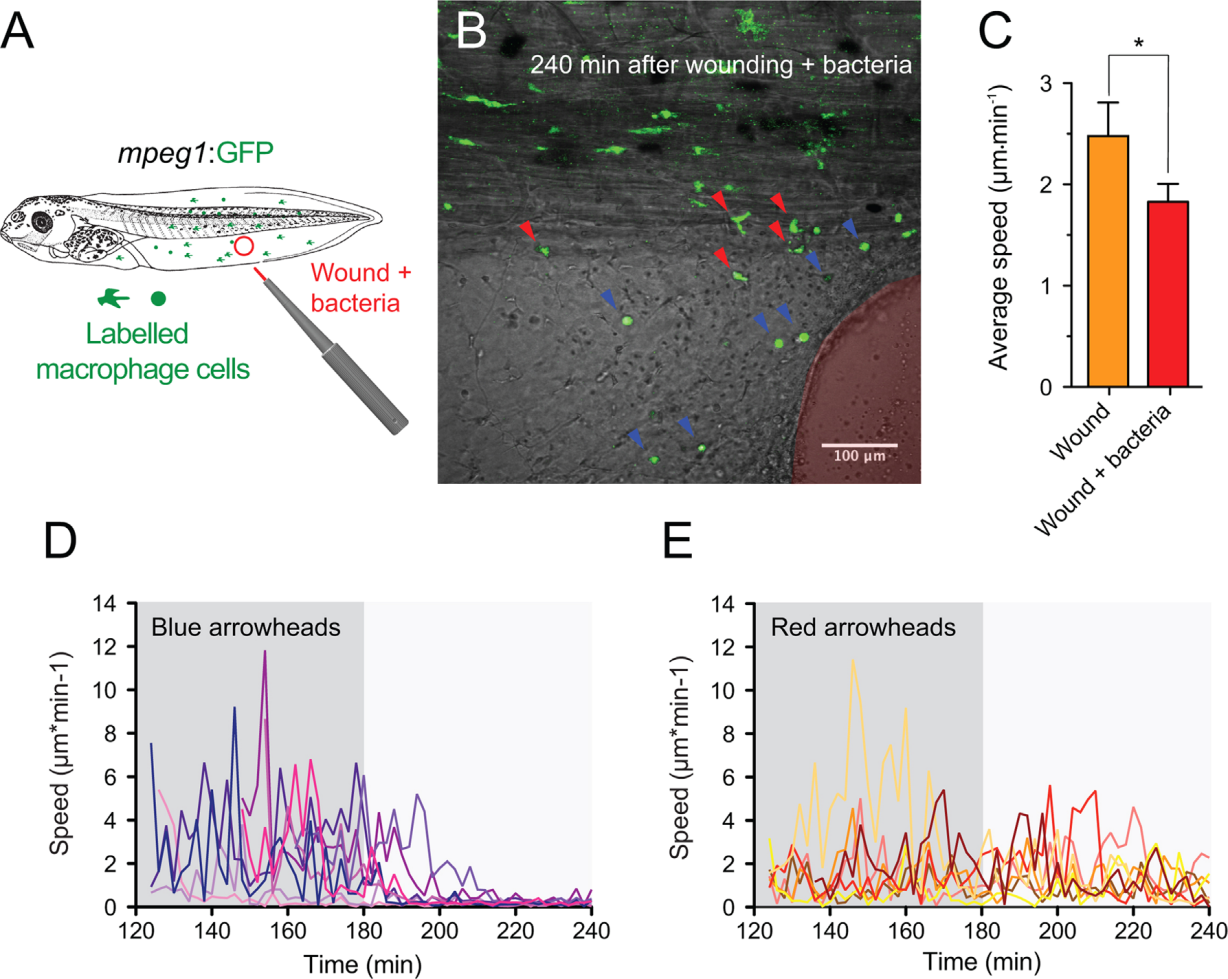
Macrophage response to bacterially infected wounds. (A) Schematic representation of the wounding assay plus bacteria infection in *mpeg1*:GFP transgenic *Xenopus* larva. The puncher was loaded with live *E. coli* (red tip) before generating the excisional wounds. (B) Single-channel confocal projection merged with the best in focus transmitted light image of the infected wound. The excised tissue area is highlighted in red. GFP+ cells (Macrophage-like) that stopped at the infected wound margin (blue arrowheads) and at the periphery (red arrowheads) are also indicated. See supplementary video 13, associated with this panel. (C) Average speed was calculated from the tracks of mpeg+ cell from at least 3 different experiments for each condition (3 h after wounding). Mann–Whitney *U*-test, * *p*<0.05. (D) Point speeds of migration (solid lines) were traced for the cells pointed with blue arrowheads in the panel B. (E) Point speeds of migration (solid lines) were traced for the cells denoted with red arrowheads in the panel B.

Both neutrophils and macrophages are critically involved during the early stages of bacterial infection (Kimbrell and Beutler, 2001). To examine the response of these immune cell effectors to localized bacterial infection in real time *in vivo*, we modified our strategy. We first monitored the response of *mpeg1*:GFP+ cells in transgenic larvae by performing a small lesion in the fin as before, but using a biopsy puncher loaded with live bacteria (*E. coli*) (Fig. 8A). As in aseptic wounds, infected wounds induced a rapid recruitment of mpeg+ cells within the first hour post injury. We did not find a significant difference in the time required for cells to start moving toward the site of injury, when comparing aseptic wounds *versus* infected wounds (data not shown). Interestingly, however, a subset of mpeg+ cells recruited to the infected site slowed down and adopted a rounded shape (Fig. 8B, blue arrowheads; Supplementary video 13), whereas other mpeg+ cells retained active migration (Fig. 8B, red arrowheads). To further analyze the motility of the mpeg+ cells after infected injury, we determined the average speed of the macrophage-like cells in 3 independent experiments. We found that bacterial stimuli significantly reduced the average speed of mpeg+ cells after they were recruited to the wound site (Fig. 8C). The slowdown in motility did not affect the preferential direction of movement towards the injury site (data not shown). The behavior of these stalling cells was similar to that exhibited by the phagocytic macrophage-like cells we described in Fig. 3, with peaks of high point speed before to reach the target to then slow down and finally stop (Fig. 8D). Mpeg+ cells located in the periphery of the infected wound site did not show this stalling property during the same time period of observation, indicating a specific macrophage-like activity at the infected site (Fig. 8E).

**Fig. 9:**
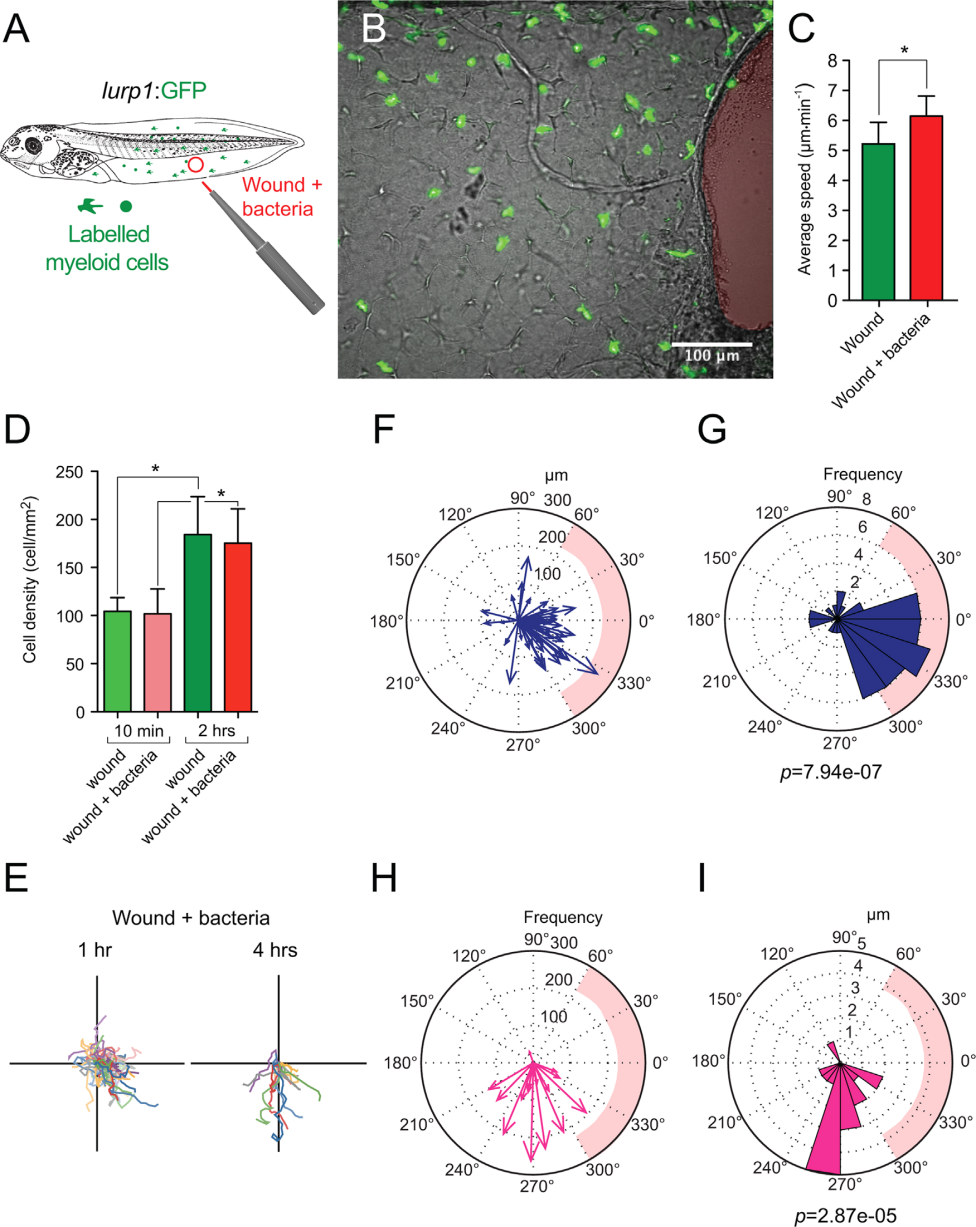
Granulocyte response to bacterially infected wounds. (A) Schematic representation of the wounding assay plus bacteria infection in *lurp1*:GFP transgenic *Xenopus* larva. The puncher was loaded with live *E. coli* (red tip) before generating the excisional wounds. (B) Single-channel confocal projection merged with the best in focus transmitted light image of the infected wound. Granulocyte-like cells are labeled in green and the excised tissue area is highlighted in red. See supplementary video 14, associated with this panel. (C) Average speed was calculated from the tracks of *lurp*+ cells from at least 3 different experiments for each condition (1 h after wounding). Mann–Whitney *U*-test, *p<0.05. (E) The tracks obtained from the *lurp*+ cells at 1 h and 4 h after wounding were plotted forcing a common origin in a quadrant mesh. (F) and (G) The magnitude and the angles of the displacement vectors obtained from the *lurp*+ cell tracks 1 h after biopsy punch-wounding assay were plotted in rose charts (MatLab). The location of the wound site is denoted by a pink overshadow. Rayleigh test (CircStat for MatLab, The MatWorks) *p*-value is also indicated. (H) and (I) The magnitude and the angles of the displacement vectors obtained from the *lurp*+ cell tracks 4 h after biopsy punch-wounding assay were plotted in rose charts (MatLab). The location of the wound site is denoted by a pink overshadow. Rayleigh test (CircStat for MatLab, The MatWorks) *p*-value is also indicated.

To compare the response of putative granulocytes during localized bacterial infection, we used the *lurp1*:GFP transgenic line (Fig. 9A). In contrast to mpeg+ cells, lurp+ cells with morphological features of granulocytes exhibited an active behavior with a mild but significant increase in the average speed a few hours following the bacterial-wound stimuli, (Fig. 9B; Supplemental video 14, and Fig. 9C). As we observed for the putative macrophage cells, bacterial infection did not change significantly the time required for cell activation in the lurp+ population (data not shown). Although the overall number of myeloid cells recruited to the infected wound site increased over the first few hours, It was not significantly different to the number recruited to aseptic wounds (Fig. 9D). This suggests that bacterial infection did not markedly affect the strength of the recruitment signals at the injury site. Lurp+ cells migrated towards the wound site within the first couple of hours (Fig. 9E, left hand side, Fig. 9F and G) reaching the edge and crawling in close vicinity of the wound margin in a similar manner than in sterile wound situation. Nevertheless, after 4 h of bacterial presence, granulocytes were recruited to the infected tissue several hundred micrometers away from the wound edge, reflected in the change of the preferred direction of migration (Fig. 9E, right hand side, Fig. 9H and I). This behavior suggests that there is a change in the pattern of migration in response to bacterial stimuli. Taken together, we found active and distinctive roles of macrophage-like and granulocytes-like cells at the early stages of the wound response in *Xenopus* larval stages *in vivo*.

## Discussion

In the present study, we took advantage and further adapted the amphibian *Xenopus* tadpole as a model to investigate the inflammatory response mediated by different myeloid cell types following aseptic and infected injury by time-lapse fluorescence microscopy. Similar to zebrafish and medaka larvae, *Xenopus* tadpoles are relatively small, develop externally and are transparent. As such, *Xenopus* larvae are well suited for direct microscopy observation of physiological events such as the inflammatory response. *Xenopus* tadpoles also display remarkable abilities to repair and regenerate injured tissues and appendages (Chen et al., 2014, Love et al., 2011, Slack et al., 2008). In addition, the *Xenopus* immune system is among the most extensively characterized amongst the ectothermic vertebrates (Du Pasquier et al., 1989, Robert and Cohen, 2011, Robert and Ohta, 2009). The availability and use of *Xenopus* transgenic lines that express fluorescent reporters in specific myeloid lineages, further enhance the attractiveness of *Xenopus* by permitting real time direct observation of inflammation following injury.

The first transgenic line we used was *lurp1*:GFP, which labels a large fraction of myeloid cells (Smith et al., 2002). *Lurp1*:GFP expression was initially used to characterize early transitory events of myeloid differentiation. At later larval stages and in adults, while *lurp1*:GFP expression remained confined within the myeloid lineages (*e.g.*, no expression detected in lymphocytes), we found that not all myeloid lineages were GFP+. Nevertheless, the *lurp1*:GFP transgenic tadpoles proved useful as a large fraction of cells with a morphology of monocyte/macrophages and granulocytes (mainly neutrophil-like) were labeled by the transgene in the larvae.

The other fluorescent reporter system we used was based on the *mpeg1* promoter, which had been previously used in zebrafish to label macrophages (Ellett et al., 2011). Similar to zebrafish, transgenic *mpeg1*:mCherry *Xenopus* tadpoles displayed fluorescent cells with monocytic/macrophage characteristics. Importantly, the transgene was not expressed in cells displaying granulocytic morphology or behavior. Double transgenic (*lurp1*:GFP/*mpeg1*:mCherry) tadpoles contained a subset of double positive cells (monocytes/macrophages) and single *lurp1*:GFP positive (granulocytes) cells, but we also found a minor fraction of single *mpeg1*+ cells with monocytic/macrophage morphology and behavior. This suggests that *Xenopus* larvae contain diverse populations of macrophage lineages. Furthermore, this finding shows that the *lurp1*:GFP transgene does not mark all myeloid lineages. Further studies will be needed to unravel the various myeloid subtypes that are present in *Xenopus* larvae.

We were able to follow the distinct behaviors of the monocyte/macrophage and granulocyte cells using the *lurp1*:GFP transgenic and double transgenic *lurp1*:GFP/*mpeg1*:mCherry lines. At the stage of development we performed the experiments (stage 46–47 or 4–5 days post-fertilization), the adaptive immune system was not yet fully mature (Du Pasquier et al., 1989). At this stage, the thymus is not yet organized into a cortex and medulla, and the spleen is mainly hematopoietic (*i.e.*, very few lymphocytes present) (Du Pasquier and Flajnik, 1990, Robert and Cohen, 1998). In addition, B cells still need to rearrange their immunoglobulin genes and express their Light chains (Mussmann et al., 1998). Therefore, *Xenopus* tadpoles at this developmental stage are well suited for investigating the innate immune response in real time in the absence of the adaptive immune response.

Our study indicates that *Xenopus* larvae, like fish larvae, have primarily two main types of innate immune cell effectors within the myeloid lineage, which are distinguishable by differences in their motility behaviors (Ellett et al., 2011, Grabher et al., 2007, Gray et al., 2011). As in higher vertebrates, including humans, we found that macrophages are relatively large cells, which move by extending multiple lamellipodia, with low average migration speeds of 1–2 μm/min and peak velocity up 10 μm after stimulation (Pixley, 2012, Van Goethem et al., 2010). The main difference we observe compared to mammals was the higher density of tissue resident macrophages. In murine skin, macrophage density is approximately 2 cells/mm^2^ (DiPietro et al., 1995), whereas we found that the *Xenopus* larvae tail has tissue resident macrophages in the range of 30 cells/mm^2^. This could be one of the factors that facilitate wound repair and regeneration in *Xenopus* larvae since macrophages play an active role in tissue remodeling, especially during *Xenopus* metamorphosis (Mescher et al., 2013, Nishikawa et al., 1998). In addition, we observed active neutrophil/granulocyte clearing up by macrophages-like cells at the injury site. This behavior, which has been well described in mammals (Fadel and Kagan, 2003, Gordy et al., 2011) may also contribute to the exceptional would healing capabilities of *Xenopus larvae*. The other main cell type we found were granulocytes, which were much smaller, more compact and had considerably higher motility rates in response to localized injury. Unlike the macrophages, these cells moved by projecting a single or very few lamellipodia. The average speed we measured for neutrophils ranged from 3 to 5 μm/min with peak velocities of to 20 μm/min. Thus, the morphology and motility behaviors we detected for these myeloid lineages were similar to those found in mammals, including humans (Ambravaneswaran et al., 2010, Entschladen et al., 2005, Kreisel et al., 2010).

While motility of myeloid cells upon wounding has been largely examined during sterile or aseptic conditions, less attention has been paid to cell behavior in the context of infected wounds. While, as expected, granulocytes/neutrophils are the first responders to injury, our observations revealed a complex behavior in macrophages, which display an erratic behavior in the presence of infected wounds. This type of motility is consistent with probing and/or engulfing bacteria at the infected site. Although it remains to be shown that these macrophages internalize infecting bacteria when they stutter, previous studies in *Xenopus* during early embryogenesis has shown that primitive myeloid cells are capable of internalizing bacteria efficiently *in vivo* (Chen et al., 2009).

In summary this study found that *Xenopus* larvae is an excellent system for monitoring the inflammatory response following wounding and infection in an *in vivo* context using time-lapse fluorescence microsopy. Importantly, the inflammatory response present in the larvae was similar to that seen in human patients following injury, including complex behaviors, such as leukocyte extravasation. Further studies are required to determine the extent of the complexity of the inflammatory response in this model, including whether different subsets of neutrophils and macrophages exist in this model. In particular, it is not yet known whether *Xenopus* contain both M1 and M2 macrophages and if so, whether the ratios of these myeloid subtypes change during the ontogeny of the animal or whether they mimic those seen in humans. Further exploration of such studies will help elucidate whether the quality of the inflammatory response is important in determining the quality of the reparative and/or regenerative response following injury in pre *versus* post-metamorphic *Xenopus* animals.

## Figures and Tables

**Video 1:**
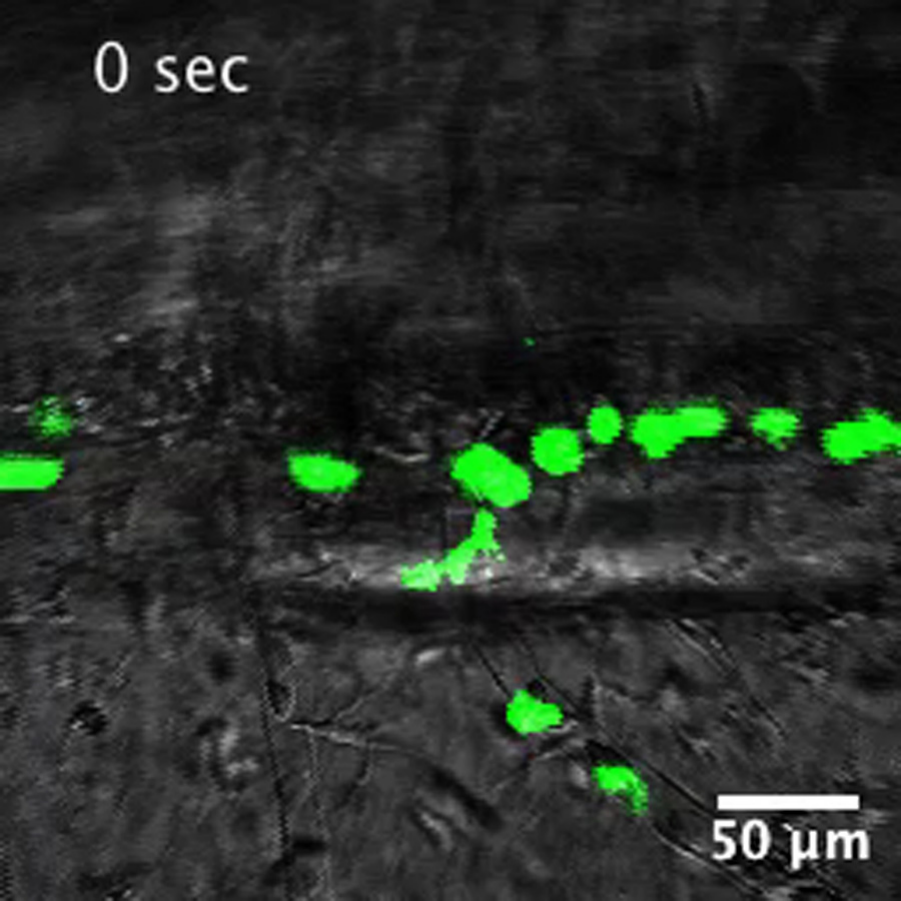
Heterogeneity of myeloid cell populations in *X. laevis* larvae: myeloid cells roll along the endothelium. Transmitted light and GFP fluorescent emission merged time-lapse of a *lurp:GFP* larva at the dorsal longitudinal anatomizing vessel (DLAV). Some of the GFP+ cells are free in circulation and others roll along the endothelial wall. A video clip is available online. Supplementary material related to this article can be found online at doi:10.1016/j.ydbio.2015.03.008.

**Video 2:**
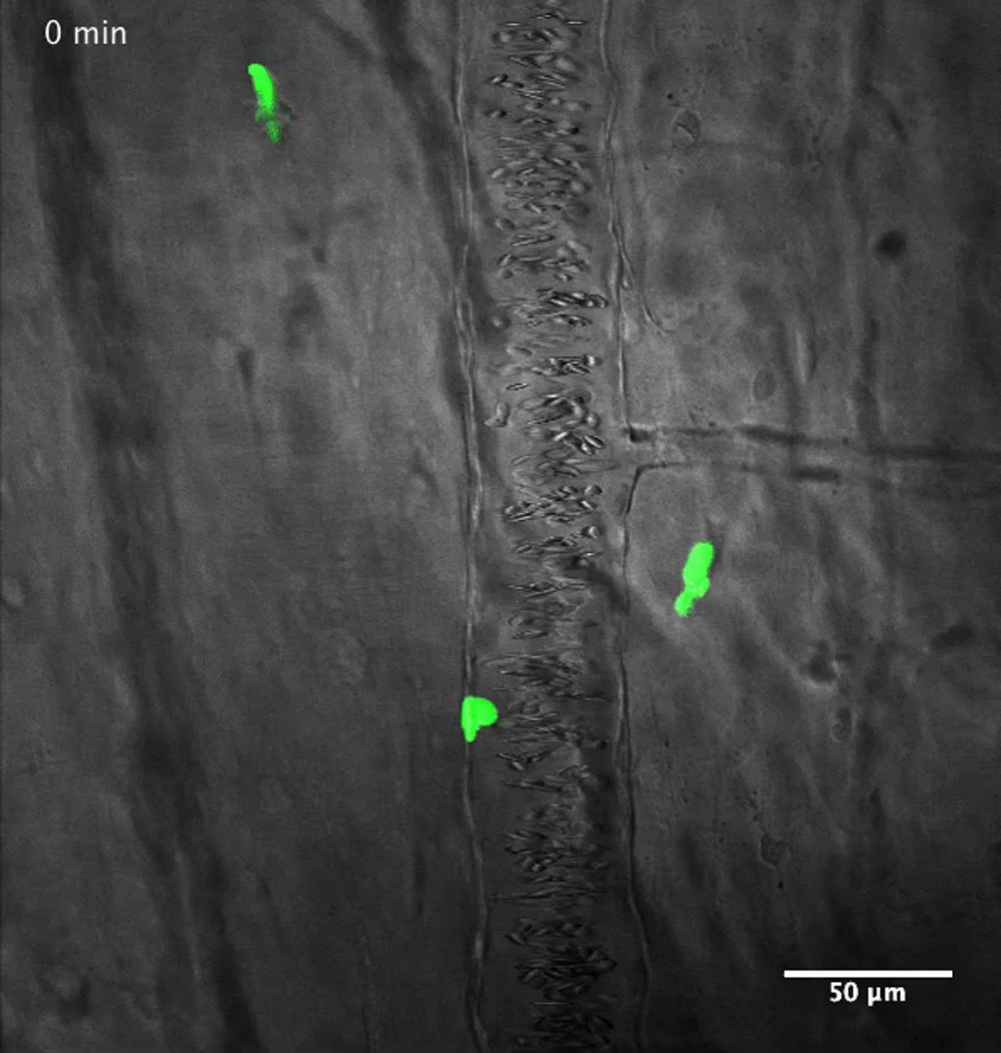
Heterogeneity of myeloid cell populations in *X. laevis* larvae**:** myeloid cells extravasation. Transmitted light and GFP fluorescent emission merged time-lapse of a capillary in a *lurp:GFP* larva after a short-term UV light exposure. GFP+ cells in circulation are accumulated at the damage area and some of them extravasate across the endothelium. A video clip is available online. Supplementary material related to this article can be found online at doi:10.1016/j.ydbio.2015.03.008.

**Video 3:**
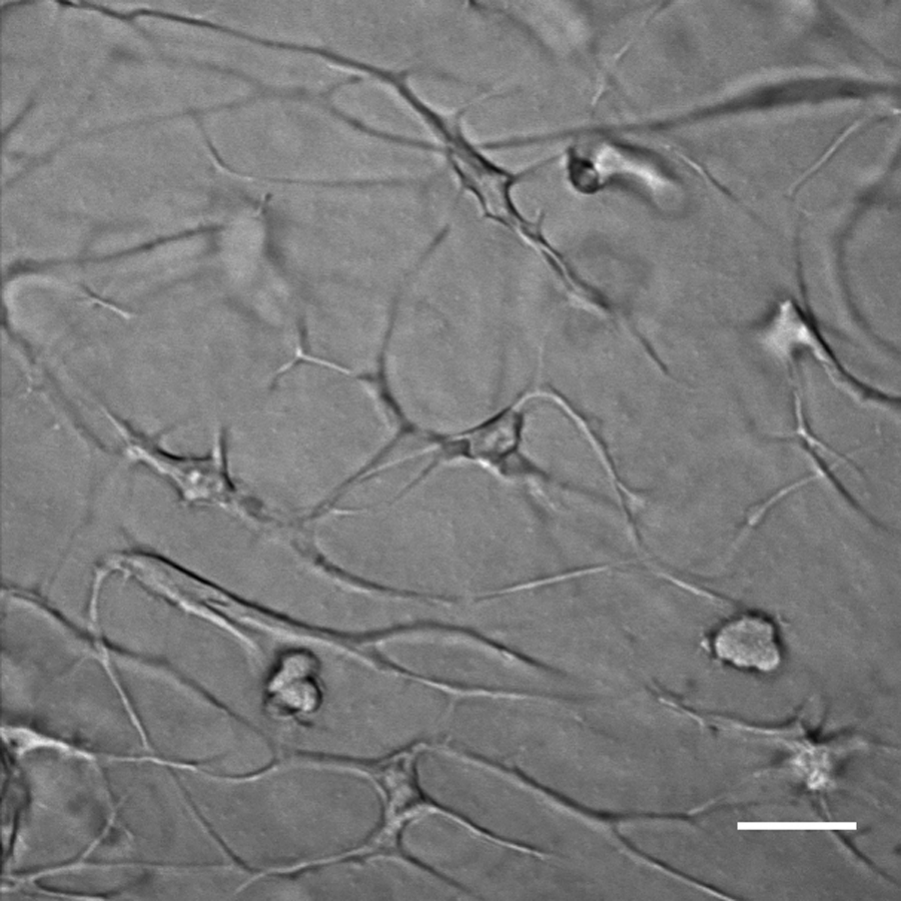
Heterogeneity of myeloid cell populations in *X. laevis* larvae: Granulocytes-like cells project lamellipodia in the direction of movement. Transmitted light time-lapse at the ventral fin of a *Xenopus* larva showing *in vivo* cell migration within the tissue. Note forward projecting lamellipodia as the cells move. A video clip is available online. Supplementary material related to this article can be found online at doi:10.1016/j.ydbio.2015.03.008.

**Video 4:**
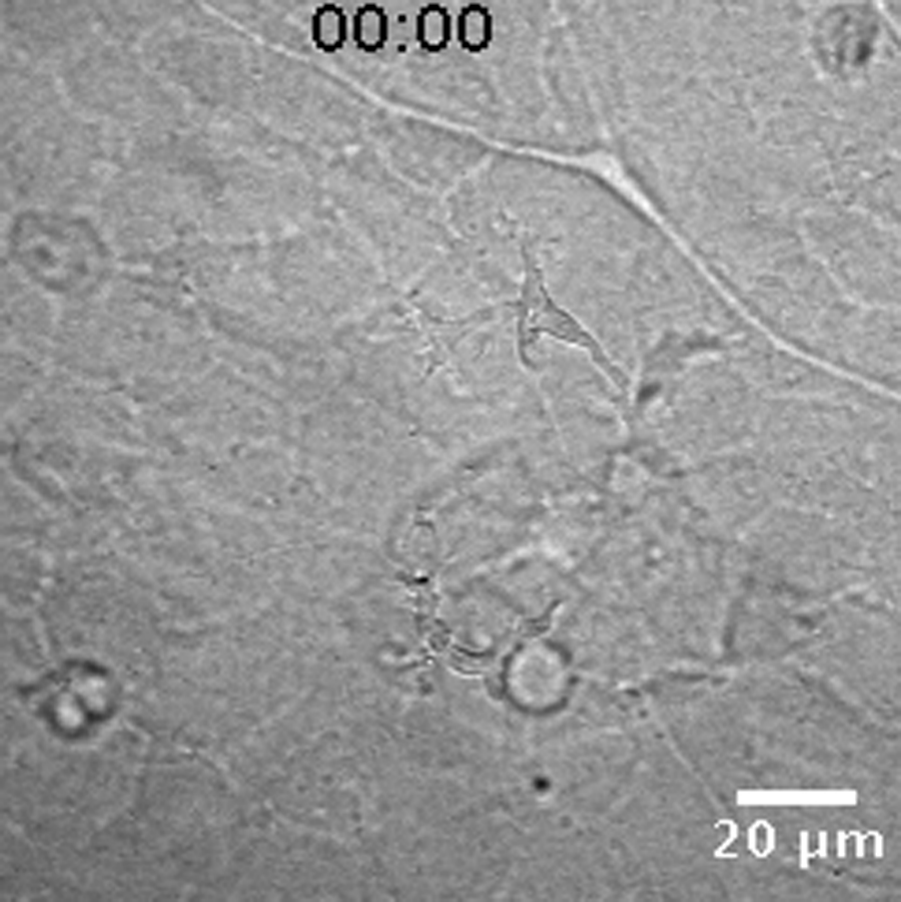
Heterogeneity of myeloid cell populations in *X. laevis* larvae: macrophage-like cells project lamellipodia in the direction of movement. Transmitted light video (30 min time lapse) at the ventral fin of a *mpeg1*:GFP transgenic *Xenopus* larva showing macrophage-like cell migration within the tissue. Note several lamellipodia projections form while the cells move. A video clip is available online. Supplementary material related to this article can be found online at doi:10.1016/j.ydbio.2015.03.008.

**Video 5:**
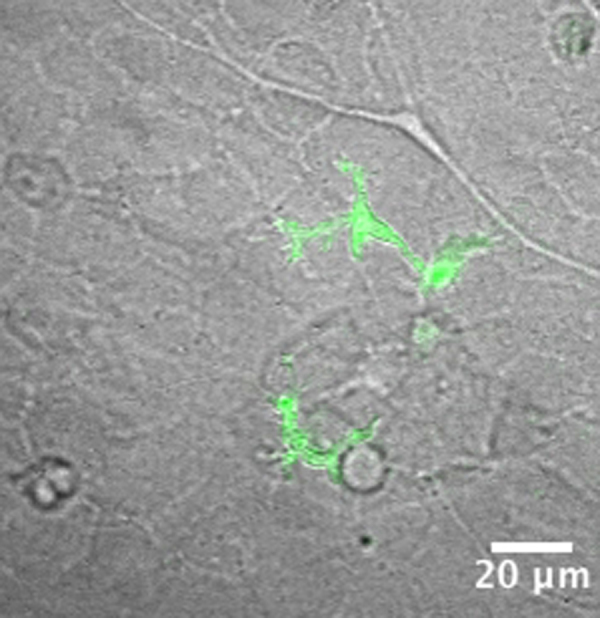
Heterogeneity of myeloid cell populations in *X. laevis* larvae: macrophagess-like cells project lamellipodia in the direction of movement. Transmitted light and GFP fluorescent emission merged time-lapse version of the same video shown in video 4. A video clip is available online. Supplementary material related to this article can be found online at doi:10.1016/j.ydbio.2015.03.008.

**Video 6:**
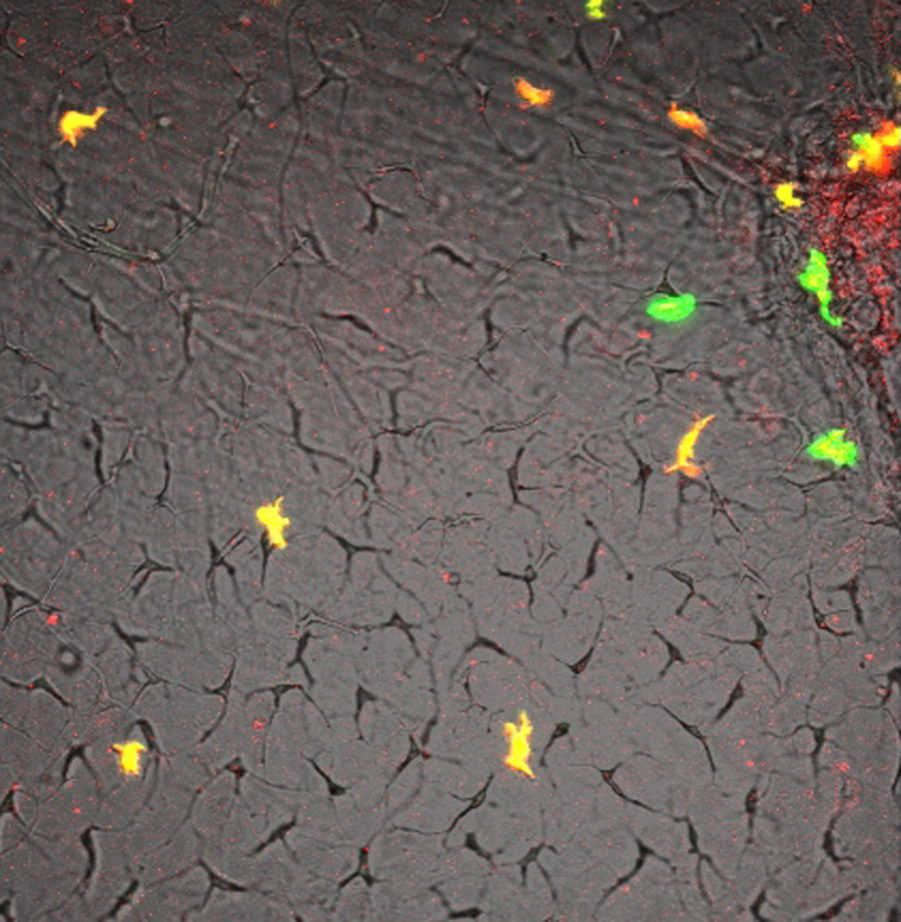
Heterogeneity of myeloid cell populations in *X. laevis* larvae: cell migration toward the wound site: 0.3 mm biopsy punch assay in the fin of a *lurp*:GFP/*mpeg1*:Cherry double transgenic *Xenopus* larva. Green cells behave like granulocytes and yellow/red cells behave like macrophages. The movie reflects 3 h of cell migration starting 5 min after injury located at the top right of the movie. 10 confocal planes (Z-stack) were taken each 2 min, flattened down and projected as a continuous time-lapse. A video clip is available online. Supplementary material related to this article can be found online at doi:10.1016/j.ydbio.2015.03.008.

**Video 7:**
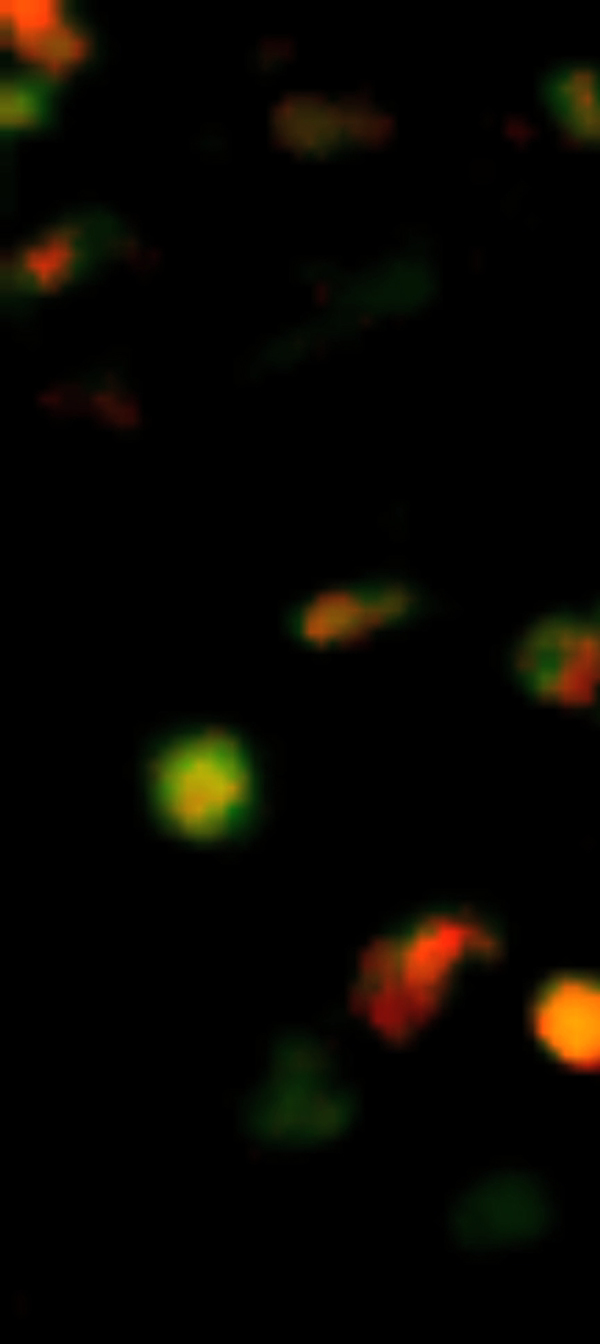
Tracing myeloid cell migration: macrophage-like cell. Macrophage engulfs a necrotic/apoptotic GFP+ granulocyte near the wound site at the bottom of the video. Time-lapse of myeloid behavior at the wound site 24 h after wounding in the fin of a *lurp*:GFP/*mpeg1*:Cherry double transgenic *Xenopus* larva. Green cells are neutrophil-like cells and yellow/red cells are macrophage-like cells. A video clip is available online. Supplementary material related to this article can be found online at doi:10.1016/j.ydbio.2015.03.008.

**Video 8:**
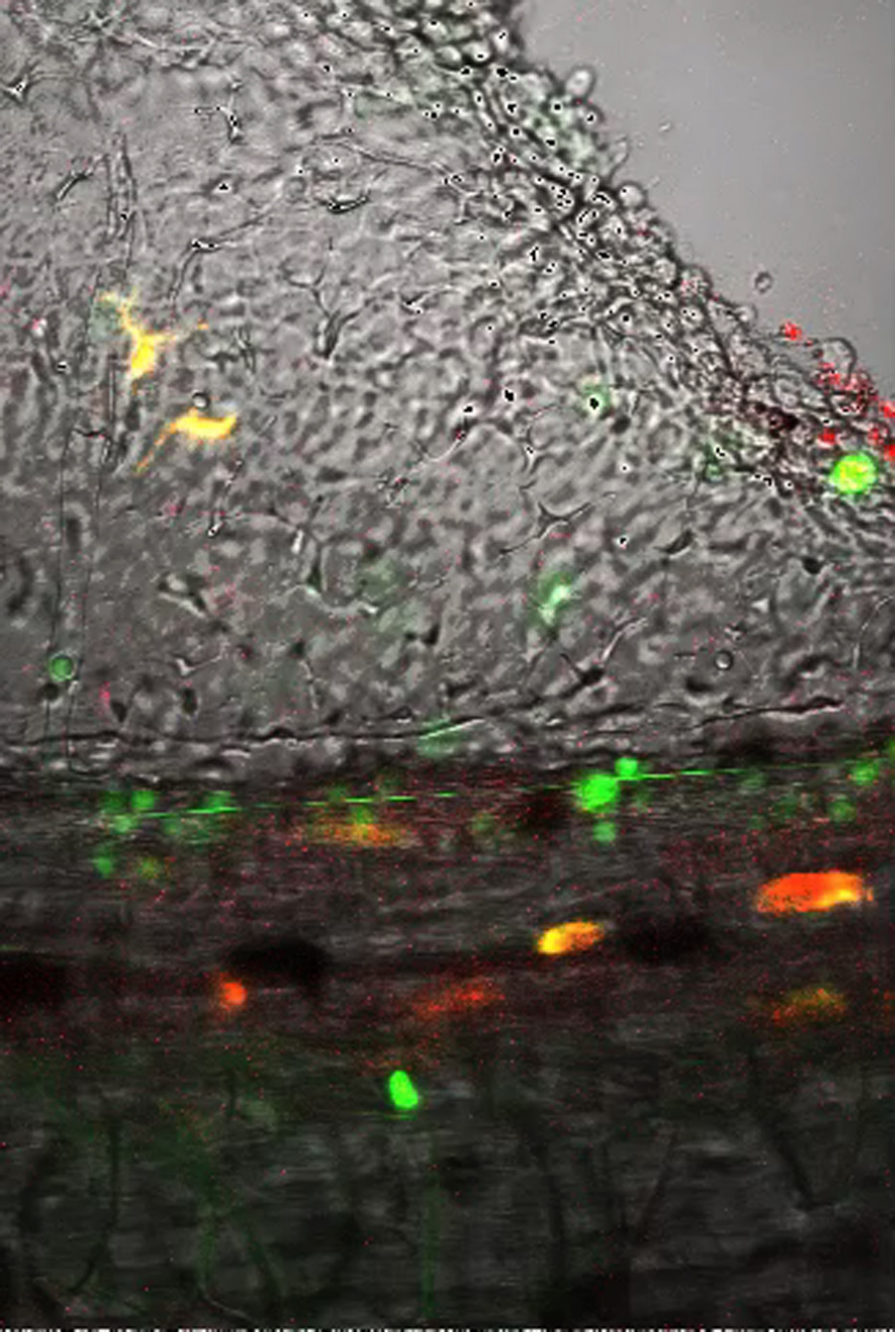
Tracing myeloid cell migration: neutrophil-like cell. 0.3 mm biopsy punch assay in the fin of *lurp*:GFP/*mpeg1*:Cherry a double transgenic *Xenopus* larva. Green cells are granulocytes-like cells. The movie reflects 144 min of cell migration starting 10 min after injury located at the top right of the movie. 10 confocal planes (Z-stack) were taken each 2 min, flattened down and projected as a continuous time-lapse. A video clip is available online. Supplementary material related to this article can be found online at doi:10.1016/j.ydbio.2015.03.008.

**Video 9:**
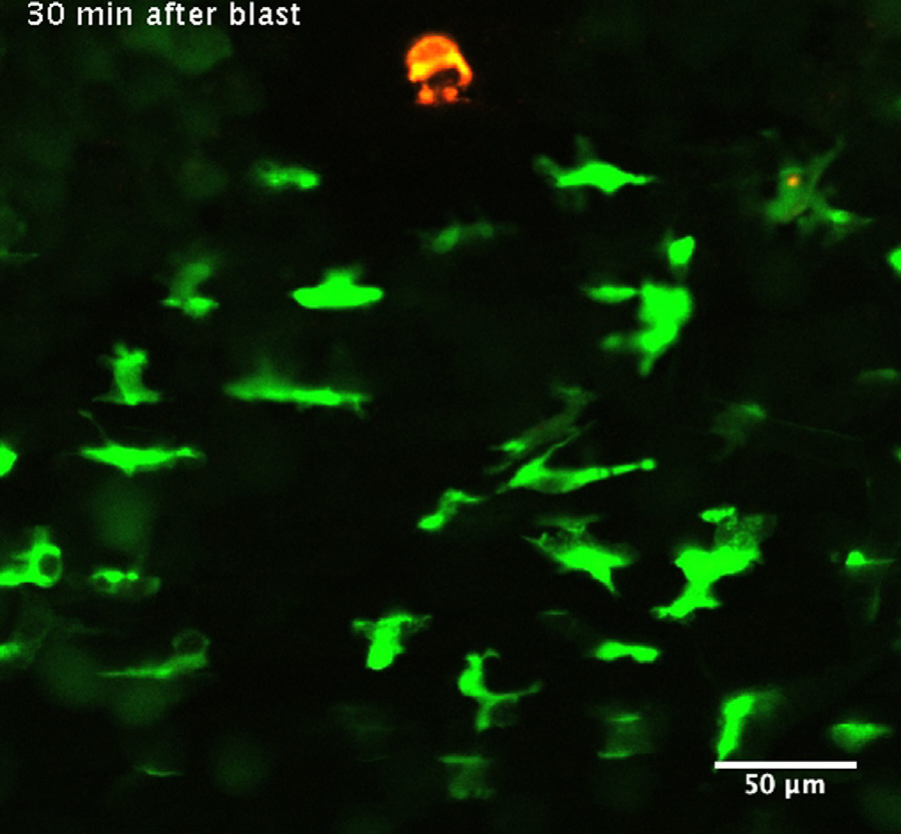
Real-time responses of myeloid cells to mechanical injury: LASER melanophore ablation. Dual channel confocal time lapse of an *lurp*:GFP *Xenopus* larva after LASER melanophore ablation injury. Frames were acquired each 30 s starting 30 min after the LASER blast. Melanophore cell debris produces a red fluorescence that can be seeing at the top of the clip. GFP+ actively migrate towards the injury site. A video clip is available online. Supplementary material related to this article can be found online at doi:10.1016/j.ydbio.2015.03.008.

**Video 10:**
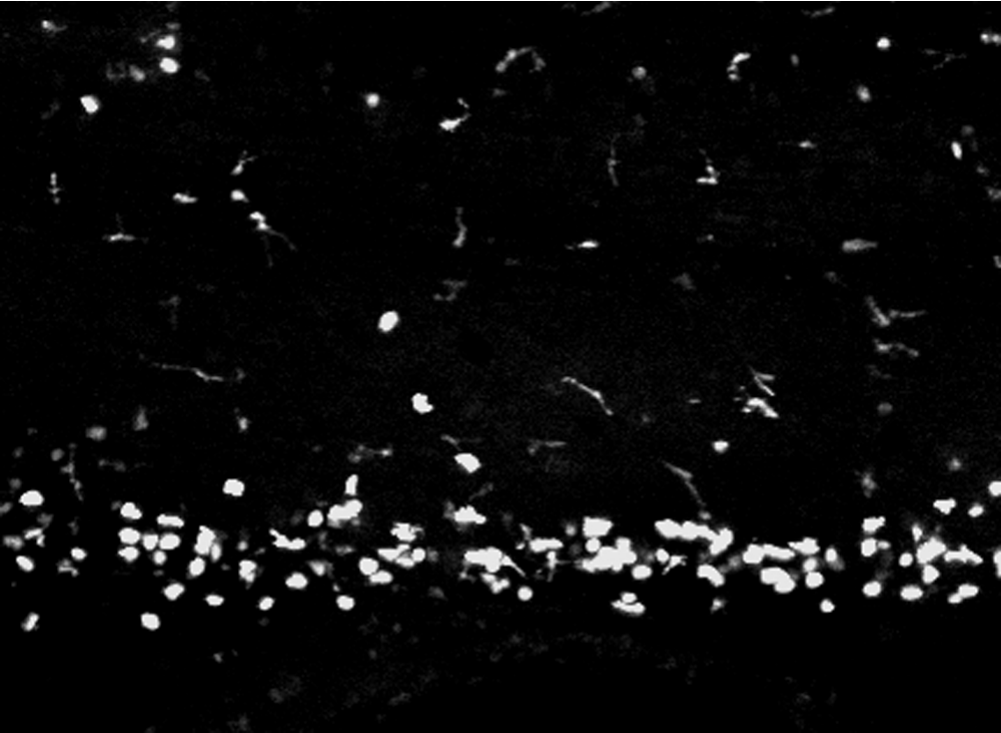
Myeloid cells migrate preferentially towards the wound site after injury. 0.3 mm biopsy punch assay in the fin of a *lurp*:GFP transgenic *Xenopus* larva. The movie reflects 9 h of cell migration starting 15 min after injury located at the bottom of the movie. 5 confocal planes (Z-stack) were taken each 2 min, flattened down and projected as a continuous time-lapse. A video clip is available online. Supplementary material related to this article can be found online at doi:10.1016/j.ydbio.2015.03.008.

**Video 11:**
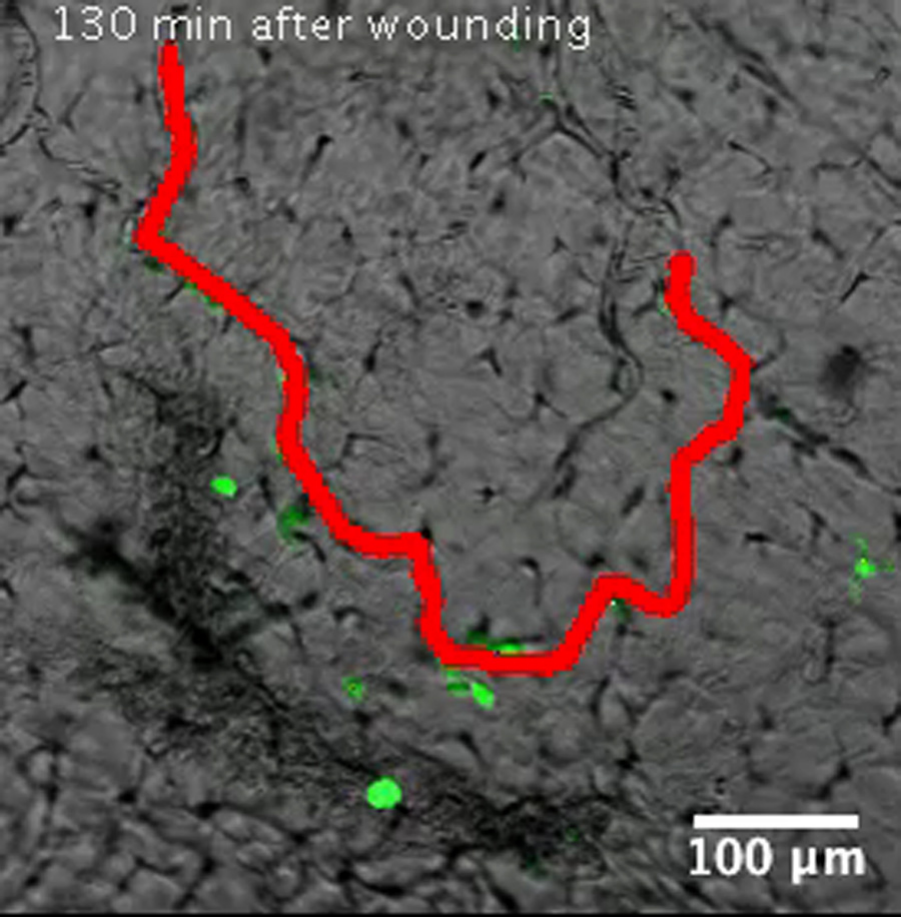
Leukocyte extravasation of myeloid cells in response to mechanical wounds: capillary far from wound site. Transmitted light and GFP fluorescent emission merged time-lapse of a *lurp*:GFP transgenic *Xenopus* larva 130 min after a biopsy punch wound, located at the right of the movie (about 1 mm out of the field of view). A colored red mask has been created to highlight a blood vessel (first frame). Multiple colored path lines denote the calculated tracks for the GFP+ cells that extravasated from the capillary. A video clip is available online. Supplementary material related to this article can be found online at doi:10.1016/j.ydbio.2015.03.008.

**Video 12:**
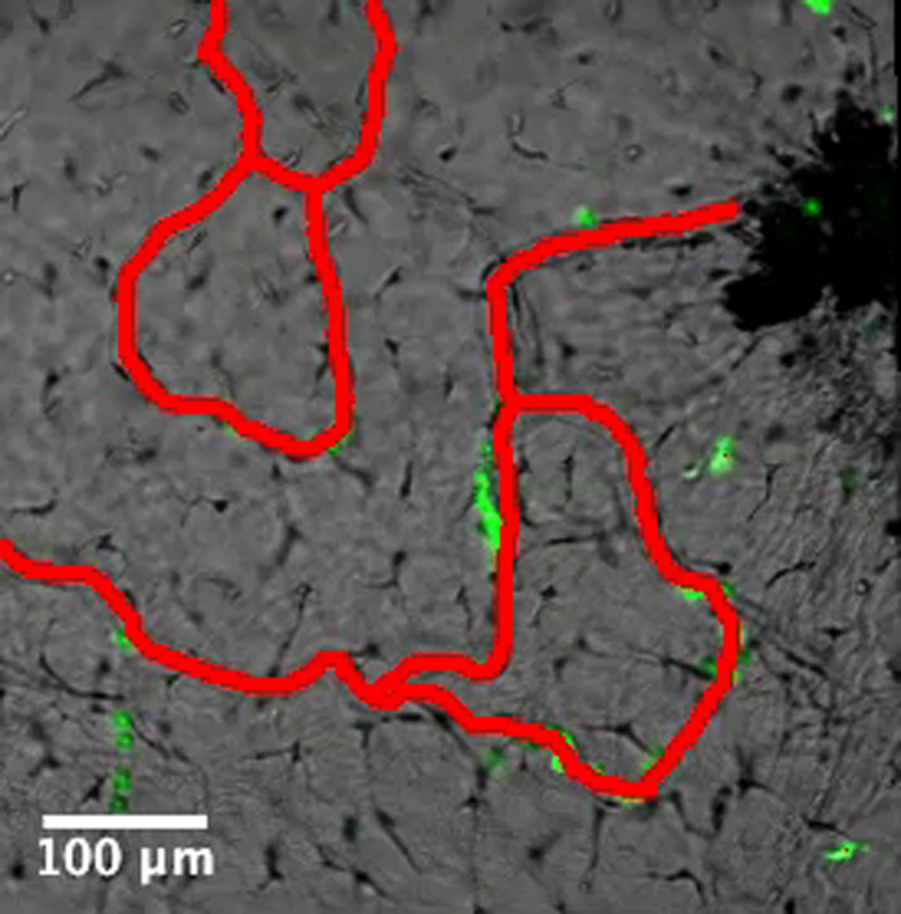
Leukocyte extravasation of myeloid cells in response to mechanical wounds: capillary near the wound site. Transmitted light and GFP fluorescent emission merged time-lapse of a *lurp*:GFP transgenic *Xenopus* larva 130 min after a biopsy punch wound, located at the right of the movie. Colored red masks (first frame) have been created to highlight blood vessels within 0.5 mm of the wound site. Multiple colored path lines denote the calculated tracks for the GFP+ cells that extravasated from the capillaries. A video clip is available online. Supplementary material related to this article can be found online at doi:10.1016/j.ydbio.2015.03.008.

**Video 13:**
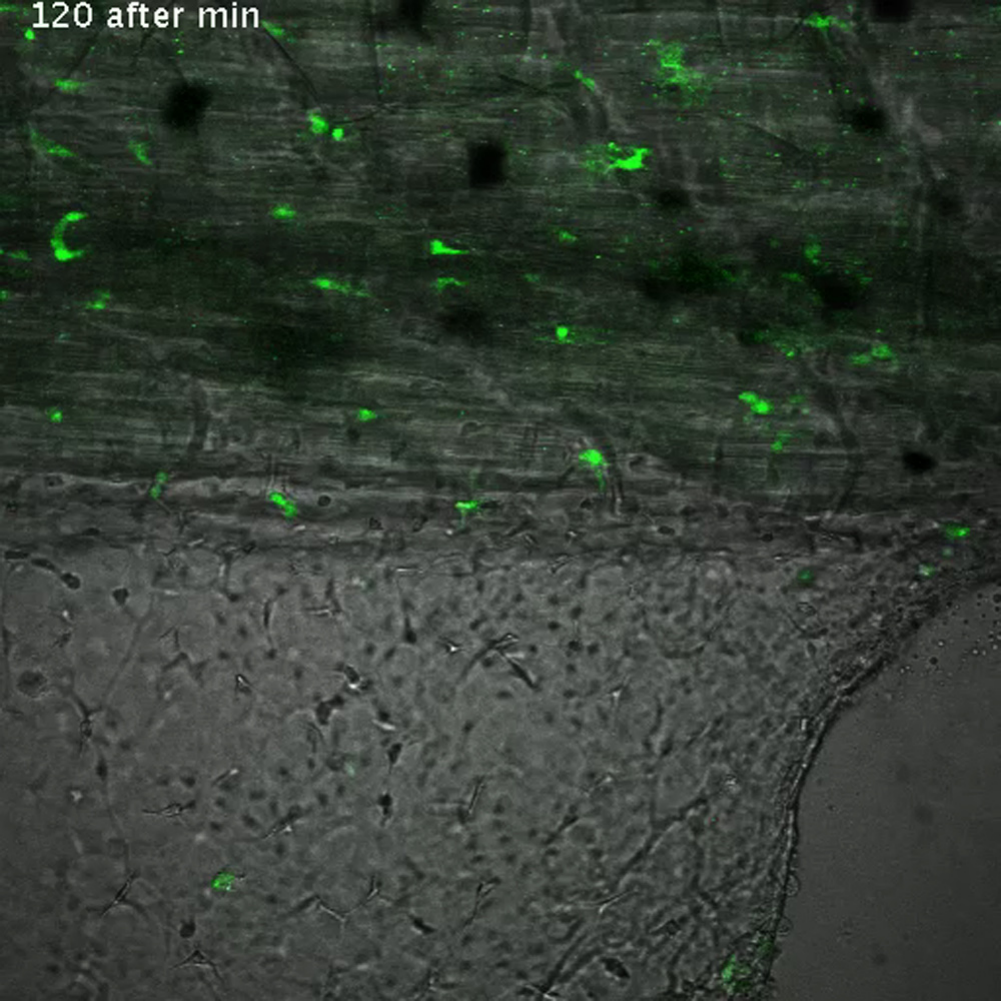
Macrophage response to bacterially infected wounds. Wound assay plus bacteria infection in the fin of a *mpeg1*:GFP transgenic *Xenopus* larva. The wound is located at the bottom right of the movie. 10 confocal planes (Z-stack) were taken each 2 min, flattened down and projected as a continuous time-lapse. A video clip is available online. Supplementary material related to this article can be found online at doi:10.1016/j.ydbio.2015.03.008.

**Video 14:**
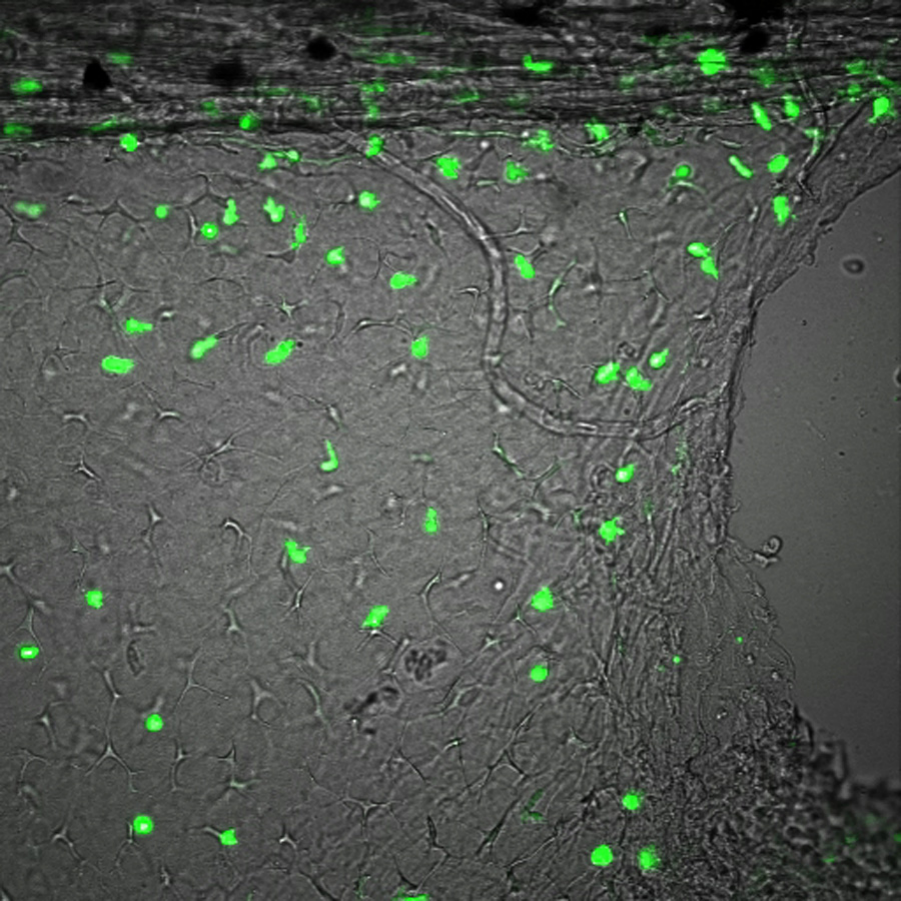
Granulocyte response to bacterially infected wounds. Wound assay plus bacteria infection in the fin of a *lurp*:GFP transgenic *Xenopus* larvae. The wound is located at the right of the movie. 10 confocal planes (Z-stack) were taken each 2 min, flattened down and projected as a continuous time-lapse. A video clip is available online. Supplementary material related to this article can be found online at doi:10.1016/j.ydbio.2015.03.008.
